# Evaluating Large Language Model–Derived Outputs as Psychological Measures: A Five-Domain Psychometric Framework With Application to Daily Stress

**DOI:** 10.2196/102768

**Published:** 2026-10-15

**Authors:** Inyong Jeong, Soohyun Park, Min Jhon, Dae-Kwang Kim, Jin-Hyun Park, Yeongmin Kim, MyeongGyun Jang, Jae-Min Kim, Ju-wan Kim, Hwamin Lee

**Affiliations:** 1 Department of Biomedical Informatics, Korea University College of Medicine, Seoul, Republic of Korea, 82 2-3407-2099; 2 Chronobiology Institute, Korea University, Seoul, Republic of Korea; 3 Department of Psychiatry, Chonnam National University Medical School, Gwangju, Republic of Korea

**Keywords:** psychometrics, reliability and validity, large language models, natural language processing, patient-reported outcome measures, ecological momentary assessment, occupational stress, digital health, psychometric evaluation, generative artificial intelligence, within-person measurement, lexicon-based analysis, voice narratives, mental health

## Abstract

**Background:**

Large language models (LLMs) are increasingly used to quantify psychological constructs from natural language, yet their outputs are typically evaluated only as predictions of an external label. When that label is itself imperfect, optimizing prediction conflates label agreement with construct measurement. No study has applied a multidomain measurement-oriented evaluation to LLM-derived psychological outputs.

**Objective:**

We applied 5 psychometric evaluation domains to LLM-derived outputs to test whether measurement-oriented evaluation surfaces properties that prediction-oriented evaluation does not: extraction repeatability, convergent validity with a lexicon-based tool, ecological concurrent validity decomposed into within-person and between-person components, incremental value in a 4-arm comparison with that tool, and method-blinded expert evaluation with structured error attribution.

**Methods:**

Thirteen linguistic features were extracted by GPT-5.2 (OpenAI) from 3556 person-days of daily voice narratives collected from 279 call center workers over 15 days via ecological momentary assessment. Features were evaluated across the 5 domains, with Linguistic Inquiry and Word Count, version 22, as the lexicon-based comparator. Multimodel robustness was assessed by re-extracting all features with Google Gemini 2.5 Flash.

**Results:**

Features showed high repeatability (mean intraclass correlation coefficient=0.987), construct-dependent convergent validity (Spearman ρ=0.08 to 0.79), and within-person tracking of same-day stress (negative emotion β=0.68). In a 4-arm comparison (baseline, baseline plus lexicon features, baseline plus LLM-derived features, and baseline plus both), LLM features increased explained variance in daily stress from 13.2% to 30.4%, versus 16.9% for the lexicon-based comparator. Among transcripts sampled for maximal method discordance, experts preferred LLM-derived profiles in 83.7% (261/312) of blinded decisions. Error analysis showed that 53% (35/66) of false-positive attributions reflected reference-label limitations, not model failures. Re-extraction with Google Gemini 2.5 Flash replicated key properties (person-level *r*=0.94). Three phenomena were visible under measurement-oriented evaluation that prediction-oriented evaluation does not typically examine: divergence between within-person and between-person effects, reference-label limitations as a dominant error source, and moderation of feature meaning by individual differences.

**Conclusions:**

Prediction metrics alone are insufficient for evaluating LLM-derived psychological measures. Measurement-oriented evaluation revealed properties, including level-dependent effects and asymmetric error attribution, that have direct implications for how LLM-based assessment studies interpret their results. For clinical use, the measure is a complement to existing screening instruments for longitudinal monitoring, not a stand-alone diagnostic.

## Introduction

Large language models (LLMs) are increasingly used to quantify psychological constructs from natural language. Recent work has shown that commercial LLMs can score personality traits from open-ended text with convergent validity comparable to established benchmarks [1], and a growing infrastructure now supports the sharing and application of language-based assessment models across constructs including depression, well-being, and implicit motives [2-4]. As these tools move from proof-of-concept to routine use, a question arises that the field has not yet addressed: how should LLM-derived outputs be evaluated when they are intended to serve as psychological measures?

Current evaluation practice treats this question as a prediction problem [5-7]. LLM outputs are scored against an external reference, typically a self-report scale, with performance indexed by classification accuracy or correlation. A review of health care LLM studies reports the same pattern: accuracy was the predominant evaluation dimension, while fairness, bias, and toxicity were rarely assessed [8]. This approach is appropriate when the reference is a well-validated criterion but becomes problematic when the reference itself has known limitations. The Perceived Stress Scale (PSS-10), for example, lacks consensus on clinical cutoff thresholds [9-11], and even scales with established thresholds such as the Patient Health Questionnaire-9 (PHQ-9) impose categorical distinctions on phenomena that are conceptually continuous. When a new tool is evaluated primarily by how well it predicts an imperfect label, label agreement is conflated with construct measurement, and the limitations of the reference propagate into the evaluation of the new tool.

The distinction between prediction and measurement is not new. Construct validity theory has long held that convergence with a single criterion is insufficient to establish that a measure captures its intended construct [12,13]. The relevant criteria are those applied to any measurement instrument: reliability under repeated administration, convergence with related measures, divergence from unrelated ones, sensitivity to theoretically expected variation, and incremental value beyond existing tools. For LLM-derived outputs specifically, two additional considerations apply. First, because the extraction process is stochastic, repeatability of feature values across identical inputs must be established before other properties can be interpreted. Second, because LLM outputs can be compared against existing lexicon-based tools such as Linguistic Inquiry and Word Count (LIWC) [14,15], the evaluation can quantify not only whether LLM features are valid, but whether they capture construct-relevant information that lexicon-based approaches miss.

Lexicon-based text analysis assigns words to predefined psychological categories and scores a document by the proportion of its words falling in each category. LIWC is the most widely used implementation and has served as the default instrument in psychological text research for two decades. Its categories are fixed, its scoring is deterministic, and its behavior is therefore fully auditable. It is also insensitive to context: negation, irony, and reported speech are scored on the same terms as direct expression, and the dictionary exists for a limited set of languages.

Several lines of work have begun to connect psychometric theory with LLM research. Alaa et al [16] argued that construct validity should guide evaluation of medical LLM benchmarks, Li et al [17] evaluated the psychological reasoning of LLMs against psychometric criteria, and Serapio-García et al [18] developed a psychometric framework for personality traits expressed by LLMs themselves. Most directly, Wright et al [1] demonstrated that LLMs can measure stable personality traits from open-ended narratives. That study answered a foundational question: whether LLMs can measure psychological constructs from text. A different question follows: when LLM-derived outputs are subjected to the multidomain evaluation criteria long applied to rating scales, do properties emerge that prediction-oriented evaluation does not detect? This question is especially demanding for psychological states, which fluctuate within individuals over short timescales and require the instrument to distinguish true within-person change from measurement noise.

We apply 5 psychometric evaluation domains to LLM-derived outputs, using daily occupational stress in call center workers as the empirical context. The 5 domains are not construct-specific; they operationalize standard psychometric criteria and can be applied whenever LLM-derived scores are intended to function as psychological measures. The data comprise 15 days of repeated daily observations from 279 call center workers, including brief stress ratings collected via ecological momentary assessment (EMA) twice per day and short voice narratives recorded 3 times per day in response to time-specific open-ended prompts. Call center work was selected because the relatively homogeneous work environment supports interpretability of feature behavior, and the elevated stress burden [19-22] provides a context where stress-related linguistic signals should be observable if the measurement approach is sufficiently sensitive. The material analyzed here is spoken: short voice narratives recorded 3 times a day in response to time-specific prompts, then transcribed. The input is elicited rather than passively captured, and momentary rather than retrospective. We also test whether feature-stress associations vary with depressive symptom severity and personality traits, and replicate key properties using a second LLM (Gemini 2.5 Flash).

We therefore ask whether measurement-oriented evaluation reveals properties of LLM-derived psychological outputs that prediction-oriented evaluation does not. To answer this, we apply 5 psychometric evaluation domains: extraction repeatability, convergent validity with an existing lexicon, ecological concurrent validity, incremental value in a 4-arm comparison, and method-blinded expert evaluation.

## Methods

### Overview

The evaluation covered five psychometric domains, each addressing a distinct measurement question: (1) extraction repeatability, (2) convergent validity with a lexicon-based tool, (3) ecological concurrent validity with within-person and between-person decomposition, (4) incremental explanatory value in a four-arm comparison, and (5) method-blinded expert evaluation with structured error attribution. The operationalization of each domain is described after the data collection procedures.

### Study Design and Participants

This prospective, multisite cohort study used a 15-day EMA design to evaluate LLM-based psycholinguistic measurement in an occupational cohort. Participants were recruited from 3 corporate call center sites and one public agency between August 3, 2023, and January 25, 2024, in a companion study conducted by the same investigators [23]. Workers were informed through workplace notices, and baseline assessment and app setup were carried out on site. All participants performed emotional labor, including civil consultation, customer service, sales, or administrative duties. Eligibility criteria were age ≥19 years, at least 6 months of employment at the current workplace, fluency in Korean, and daily smartphone access.

Prespecified analytic exclusion criteria were (1) missing baseline or follow-up PSS-10 data, (2) invariant PSS-10 responses (total score of 0 at both time points), (3) mean day-level transcript length <10 words, or (4) fewer than three completed EMA days. Of the 486 enrolled participants, 207 were excluded under these criteria: 165 for missing follow-up PSS-10 data, 18 for invariant PSS-10 responses, 15 for insufficient transcript length, and 9 for fewer than 3 completed EMA days. The largest exclusion category therefore reflects loss to follow-up rather than data quality, and the participant flow diagram is provided in Figure S1 in Multimedia Appendix 1.

At baseline, participants completed in-person surveys assessing sociodemographic characteristics, depressive symptoms, perceived stress, and personality traits, then installed the study-specific smartphone app. Over the subsequent 15 days, the EMA protocol comprised two parallel data streams: 3 voice narrative entries per day (morning, midday, and evening) and 2 stress ratings per day (midday and evening). Daily stress was calculated as the mean of available midday and evening ratings; days with both ratings missing were excluded from analyses involving daily stress. Daily language measures were derived from the 3 voice narratives concatenated at the day level. Word count was defined as whitespace-delimited tokens in this day-level transcript. Follow-up assessments occurred the day after the 15-day EMA period ended.

### Ethical Considerations

The study was approved by the Institutional Review Boards of Chonnam National University Hospital (CNUH) and Chonnam National University Hwasun Hospital (CNUHH) (approval numbers: CNUH-2023‐156 and CNUHH-2023‐118) and conducted in accordance with the Declaration of Helsinki. All participants provided written informed consent prior to enrollment. All data were stored under institutional review board (IRB)–approved data protection protocols. EMA data were not publicly available and could not have been included in any LLM training corpus. Participants received KRW 100,000 (KRW 1342=US $1 as of September 11, 2026) as compensation for completing the study protocol.

### Measures

#### Baseline and Follow-Up Assessments

At baseline, participants completed the PHQ-9 [24,25] to assess depressive symptom severity, the Big Five Inventory-10 (BFI-10) [26,27] for personality traits, and the PSS-10 [9,28] to assess baseline perceived stress. Demographic information was collected, including age, sex, marital status, religion, living arrangement, alcohol use, and smoking status. Following the baseline visit, participants completed EMA for 15 consecutive days. On the day after the EMA period ended, they returned for a second study visit and completed the follow-up PSS-10.

#### EMA Procedure

Voice narrative entries were collected 3 times per day at fixed times, each within a defined response window, with a push notification at the start of each session (Section S1 in Multimedia Appendix 1). Stress ratings were collected at midday and evening within the corresponding windows, using a single-item 11-point numeric rating scale from 0 (no stress at all) to 10 (extremely stressed).

For each voice narrative entry, participants responded to a time-specific open-ended prompt: in the morning, to describe their current mood; at midday, to describe the stress experienced during the morning; and in the evening, to describe the stress experienced during the afternoon. Responses were provided in an unrestricted spoken format with no time limit. For midday and evening assessments, the stress rating was completed after the voice recording. For analysis, the 3 daily voice transcripts were concatenated at the day level, and daily stress was the mean of available midday and evening ratings. A day was considered evaluable only when at least one stress rating and a day-level concatenated transcript of at least 10 words were both available; days with only a stress rating or only a voice transcript were excluded.

#### Voice Recording Transcription

All voice responses were transcribed manually in Vrew by trained annotators, with personally identifiable information removed. Transcription and standardization rules are given in Section S2 in Multimedia Appendix 1. Transcription quality was further checked using automated spell-checking.

#### LLM Psycholinguistic Feature Extraction

Reporting follows the TRIPOD-LLM guidelines [29]. Feature extraction used GPT-5.2 (OpenAI) at temperature 0 with structured output. GPT-5.2 was chosen for comparability with prior LLM-based psycholinguistic work, for reproducible structured output with a documented training cutoff, and for adequate Korean-language handling, which removed the need for upstream translation. EMA data were held under IRB-approved protection and were never public, so they could not have entered the model’s training corpus. For multimodel robustness, the same transcripts were re-extracted with Gemini 2.5 Flash under matched settings. The endpoint, parameters, retry rule, cost, and full extraction settings for both models are given in Sections S3-S7 in Multimedia Appendix 1.

Thirteen features were defined based on established LIWC categories [14,15,30] and occupation-relevant dimensions [31-33]: positive emotion, negative emotion, anxiety, anger, sadness, cognitive processing, tentativeness, self-focus, social references, work-related content, time pressure, somatic complaints, and coping.

Following established practice for structured prompt-based extraction in clinical and biomedical natural language processing [34-36], the system prompt instructed the model to function as an expert psycholinguistic rater and to follow four explicit principles: (1) base scores on the linguistic content of the transcript, (2) avoid unsupported inferences beyond what is expressed in the text, (3) prioritize conservative and reproducible scoring, and (4) favor lower rather than higher scores under uncertainty. Feature values ranged from 0.0 (absent) to 1.0 (dominant), with intermediate anchors provided to guide rating consistency (eg, 0.1‐0.2, minimal; 0.3‐0.4, moderate). Prompts were iteratively refined through preliminary testing on a convenience subset of transcripts not included in the analytic sample, with revisions guided by clinical plausibility of extracted scores and alignment with independent reading of the source text by the first author. Korean is a pro-drop language in which first-person pronouns are frequently omitted, and the prompt was adapted accordingly for self-focus scoring.

Feature extraction was applied to all 3570 person-day entries from the full merged dataset. Of these, 12 (0.34%) returned malformed JSON on the first API call and were successfully recovered through the automated retry procedure within the same extraction run; the remaining entries succeeded on the first call. No manual post hoc review of individual feature outputs was conducted; reproducibility was instead assessed through the repeated extraction procedure described in the next paragraph. Subsequently, the prespecified exclusion criterion of fewer than 3 completed EMA days was applied, yielding the final analytic sample of 3556 person-days from 279 participants. Inference volume, runtime, cost, and software versions are given in Section S7 in Multimedia Appendix 1.

#### Reliability

To assess repeatability, we randomly selected 200 EMA text entries and submitted each to the feature extraction pipeline 5 times without any changes to model configuration or prompts. Reliability was quantified using intraclass correlation coefficients (ICCs) from a 2-way random-effects model for absolute agreement, with text entries and repeated extraction runs as random effects. ICCs were calculated for both the average of 5 measurements [ICC(2,*k*), *k*=5] and a single measurement [ICC(2,1), derived via Spearman-Brown inverse] and interpreted using conventional benchmarks: <0.40 poor, 0.40 to 0.59 fair, 0.60 to 0.74 good, and ≥0.75 excellent [37]. Standard error of measurement and coefficient of variation (CV) were also computed.

#### Construct Validity

For each LLM-derived feature, a corresponding LIWC-22 category was identified a priori based on definitional overlap (eg, LLM “negative emotion” with LIWC “negemo”; LLM “work-related content” with LIWC “work”). The complete set of correspondences is specified in the analysis code accompanying this paper. Because LIWC does not natively support Korean, all EMA transcripts were translated into English using the same LLM used for feature extraction, and LIWC-22 was applied to the translated texts. Spearman rank-order correlations were computed between daily LLM-derived feature scores and corresponding daily LIWC categories, with 95% CIs from nonparametric bootstrap resampling and asymptotic 2-sided *P* values; false discovery rate (FDR)-BH correction was applied across the 13 features within each level. Day-level correlations were the primary analysis; participant-level mean correlations are reported in Table S1 in Multimedia Appendix 1. Zero-rates and word-count quartile sensitivity analyses (correlation strengths across quartiles, descriptive rather than formal interaction tests) were also examined. Correlation magnitudes were interpreted using empirically derived guidelines for psychological research rather than common thresholds, which overestimate medium and large effects: small (0.10), medium (0.20), large (0.30) [38], and 25th, 50th, and 75th percentiles at *r*=0.12, 0.24, and 0.41 [39]. For process-outcome correlations in applied clinical contexts, we also consulted field-specific benchmarks [40]. Under these guidelines, coefficients ≥0.30 are considered substantial in psychological research. Separately, Spearman correlations were computed between participant-level mean linguistic feature scores and PHQ-9, baseline and follow-up PSS-10, and BFI-10 personality traits.

#### Ecological Concurrent Validity

Same-day associations between linguistic features and daily perceived stress were assessed using generalized estimating equations (GEE) with an exchangeable correlation structure to account for repeated measures [41]. Linguistic feature values were decomposed into person-mean-centered deviations (within-person component) and person-level means (between-person component); both were standardized and entered simultaneously as predictors in each model [42]. All models were adjusted for age, sex, marital status, religious affiliation, living arrangement, alcohol use, smoking status, nonworking day, assessment day, baseline PHQ-9 score, Big Five personality traits, and daily word count. Multiple comparisons across the 13 linguistic features were controlled using the Benjamini-Hochberg FDR procedure [43]. As a sensitivity analysis, all GEE models were re-estimated using a first-order autoregressive correlation structure to assess robustness to the exchangeable assumption.

#### Incremental Value of LLM-Derived Features

To evaluate the incremental explanatory value of LLM-derived linguistic features beyond established psychological measures and beyond a lexicon-based linguistic comparator, we compared 4 nested models using ridge regression with 5-fold cross-validation. To prevent data leakage from repeated measures, cross-validation folds were constructed at the participant level, ensuring that all person-days from a given participant were assigned to the same fold. Within-person and between-person decomposition (person-mean centering and person-mean computation) and feature standardization were performed separately within each training fold and applied to the corresponding test fold using training-fold parameters. The same participant-level cross-validation scheme was applied to all predictive analyses described below.

The four models were (1) baseline (demographic variables, baseline PHQ-9 score, Big Five personality traits, and daily word count), (2) baseline+LIWC (baseline plus the 13 LIWC features in within-person and between-person decomposition), (3) baseline+LLM (baseline plus the 13 LLM-derived features in the same decomposition), and (4) baseline+LIWC+LLM (full). This 4-arm comparison isolates the contribution attributable to LLM-derived features from the contribution of any linguistic feature set. Model performance was evaluated using the coefficient of determination (*R*^2^), root mean square error (RMSE), and mean absolute error (MAE). Performance metrics were averaged across cross-validation folds.

#### Expert Evaluation 1: Blinded A/B Comparison

In the first phase, 3 experts (1 psychiatrist and 2 clinical psychologists) evaluated 104 EMA transcripts drawn from strata in which the LLM-derived and lexicon-derived scores diverged most. Preference rates should therefore be read as conditional on high method discordance rather than as population-level estimates. For each transcript, experts compared 2 deidentified linguistic feature profiles (model A and model B, corresponding to LLM and LIWC outputs, randomized per case) while blinded to the extraction method. We report interrater agreement using both Fleiss κ and Gwet AC1; Gwet AC1 is the primary metric due to its robustness to prevalence bias under highly skewed marginal distributions. The stratified sampling procedure, the verbatim evaluation items, and the post hoc power analysis for the 312 preference decisions are given in Section S8 in Multimedia Appendix 1.

#### Expert Evaluation 2: Structured Error Analysis

In the second phase, the 3 experts reviewed the 42 cases misclassified by the combined baseline+LLM model at the high-stress threshold (PSS-10≥19); this sample size was set by the number of misclassified cases and was not under design control. For each case, experts received the 15-day raw text transcripts, the 15-day EMA stress trajectory, LLM feature summaries (mean and CV), and the actual versus predicted labels. The primary cause was assigned to 1 of 3 predefined categories: data limitations, label limitations, or model limitations. Cases rated “indeterminable” on a given item were excluded from the denominator for that item. The 4 structured questions, the category definitions, and the evaluation procedure are given in Section S9 in Multimedia Appendix 1.

#### Predictive Validity

As a supplementary analysis outside the 5 evaluation domains, predictive validity for elevated-stress screening was examined. L2-regularized logistic regression models were trained to predict binary stress thresholds (PSS-10 ≥17 and ≥19) at follow-up. Class weighting was applied to address class imbalance. Model performance was assessed using area under the receiver operating characteristic curve (AUROC), area under the precision-recall curve, and Brier score, comparing 6 arms: baseline, LIWC alone, LLM alone, baseline+LIWC, baseline+LLM, and baseline+LIWC+LLM. The 2 stand-alone arms (LIWC alone and LLM alone) were added to the binary outcome analysis to characterize the discrimination provided by each feature set without baseline predictors.

#### Moderation by Depressive Symptoms and Personality Traits

Moderation analyses tested whether feature-stress associations varied with depressive symptom severity (PHQ-9≥10) or the Big Five personality traits. Depressive symptoms were chosen because affect-related linguistic markers may carry different meaning under elevated depressive burden (eg, positive language as defensive vs genuinely protective); personality traits were chosen because trait-level dispositions can shape habitual language use independent of state stress. For each LLM-derived feature, separate models tested interaction terms between the feature and each moderator. Because the 6 moderators represent separate families of hypotheses, FDR correction was applied within each moderator across the 26 interaction terms for that moderator.

## Results

### Sample Characteristics

The analytic sample comprised 279 participants contributing 3556 person-day observations over the 15-day EMA period. Participants performed a range of customer-support tasks involving routine inquiries, service coordination, and complaint handling. Exclusions are itemized in the Methods and in Figure S1 in Multimedia Appendix 1. Loss to follow-up therefore accounted for the majority of exclusions. Sample characteristics are summarized in Table 1. Participants had a mean age of 43.1 (SD 8.3) years, and 73.5% (205/279) were female. At baseline, 24.4% (68/279) met criteria for at least moderate depressive symptom severity (PHQ-9≥10). Mean PSS-10 scores were 18.3 (SD 6.4) at baseline and 15.1 (SD 6.2) at follow-up.

**Table 1.** Participant characteristics and EMA^a^ response metrics (n=279).

Characteristics	Values
Age (years), mean (SD)	43.1 (8.3)
Female sex, n (%)	205 (73.5)
Married, n (%)	155 (55.6)
No religion, n (%)	178 (63.8)
Living alone, n (%)	52 (18.6)
Alcohol use, n (%)	180 (64.5)
Smoking, n (%)	67 (24.0)
Baseline PHQ-9^b^ score, mean (SD)	6.3 (5.2)
Baseline PHQ-9≥10, n (%)	68 (24.4)
Baseline PSS^c^ score, mean (SD)	18.3 (6.4)
Follow-up PSS score, mean (SD)	15.1 (6.2)
EMA days completed (out of 15 study days), mean (SD)	12.7 (2.7)
Daily word count, mean (SD)	97.6 (45.4)
EMA-reported stress, first assessment day (0 to 10), mean (SD)	3.8 (2.4)
EMA-reported stress, overall (0 to 10), mean (SD)	3.0 (1.4)

a EMA: ecological momentary assessment; EMA metrics are summarized at the participant level.

b PHQ-9: Patient Health Questionnaire-9.

c PSS: Perceived Stress Scale.

Under the strict definition described in Methods (both voice transcript ≥10 words and at least one stress rating present on the same day), participants contributed a mean of 12.7 evaluable days (SD 2.7; median 14, IQR 13‐14) out of 15, corresponding to 85% (3556/4185) of person-day observations. By this definition, 81.7% (228/279) of participants completed ≥12 days and 15.1% (42/279) completed all 15 days. Day-level concatenated transcripts contained a mean of 97.6 (SD 45.4) words. Word-count quartiles (Q1: ≤58 words; Q4: ≥132 words) were used in subsequent sensitivity analyses. Mean EMA-reported stress was 3.8 (SD 2.4) on the first assessment day and 3.0 (SD 1.4) across the full EMA period (Table 1).

### Distribution and Internal Structure of LLM-Derived Features

LLM-derived features showed continuous distributions across the normalized 0‐1 scale, with mean and dispersion varying by construct (Table S2 and Figure S2 in Multimedia Appendix 1). Negative emotion, work-related content, and self-focus showed relatively higher mean levels, whereas sadness, anger, and coping showed lower mean levels. Several affective dimensions were mildly right-skewed.

Pairwise Spearman intercorrelations among the 13 features at the participant level were moderate rather than uniformly high (Figure S3 in Multimedia Appendix 1). Positive emotion was inversely correlated with negative emotion (ρ=–0.52) and weakly correlated with specific negative subcomponents. Within negative affect, anxiety showed stronger correlations with cognitive processing (ρ=0.55), tentativeness (ρ=0.65), and time pressure (ρ=0.53) than did anger or sadness. Cross-domain correlations included time pressure with work-related content (ρ=0.56) and somatic complaints with self-focus (ρ=0.51). Coping was positively correlated with positive emotion (ρ=0.49) and weakly with anxiety (ρ=0.27).

### Domain 1: Extraction Repeatability

Repeatability of LLM-based feature extraction was evaluated by processing identical text inputs 5 times under fixed model settings. All 13 features yielded ICC(2,*k*) values above 0.95 (Table 2). The mean ICC(2,*k*) was 0.987, ranging from 0.954 (self-focus) to 0.995 (positive emotion, negative emotion, anger, and work-related content). Single-extraction reliability was also excellent across all features [mean ICC(2,1)=0.942, range 0.806 to 0.975; all ≥0.75]. CV ranged from 8.4% (self-focus) to 49.4% (sadness), with higher CV at lower mean levels. Absolute measurement error remained small across features (mean standard error of measurement=0.016 on the 0‐1 scale).

**Table 2.** Reliability of LLM^a^-derived linguistic features (repeat extraction).

Linguistic feature	ICC(2,*k*)^b^	95% CI	SEM^c^ (0‐1 scale)	CV^d^ (%)
Positive emotion	0.995	0.995‐0.995	0.015	15.9
Negative emotion	0.995	0.994‐0.995	0.016	9.3
Anxiety	0.991	0.991‐0.992	0.013	23.5
Anger	0.995	0.995‐0.995	0.013	40.1
Sadness	0.991	0.991‐0.992	0.009	49.4
Cognitive processing	0.978	0.976‐0.979	0.020	15.1
Tentativeness	0.978	0.976‐0.979	0.017	20.5
Self-focus	0.954	0.952‐0.957	0.027	8.4
Social references	0.993	0.992‐0.993	0.015	25.5
Work-related content	0.995	0.995‐0.995	0.020	15.0
Time pressure	0.990	0.989‐0.990	0.015	26.7
Somatic complaints	0.994	0.994‐0.995	0.016	27.4
Coping	0.987	0.986‐0.987	0.013	24.7

a LLM: large language model.

b The intraclass correlation coefficients (ICCs) were estimated using a 2-way random effects model for absolute agreement [average measures: ICC(2,*k*), *k*=5]. ICCs and 95% CIs were computed using 2-way random-effects ANOVA mean squares, with average-measure CIs derived via the Spearman-Brown transformation of single-measure CIs.

c SEM: standard error of measurement.

d CV: coefficient of variation.

### Domain 2: Convergent Validity With LIWC

At the day level, convergence between LLM-derived features and corresponding LIWC categories varied substantially across constructs (Table 3). The strongest correlations were observed for social references (ρ=0.786) and work-related content (ρ=0.722), followed by somatic complaints and anger (both ρ=0.652) and positive emotion (ρ=0.632). Sadness showed intermediate correspondence (ρ=0.475), while negative emotion (ρ=0.378), cognitive processing (ρ=0.367), tentativeness (ρ=0.330), self-focus (ρ=0.283), and coping (ρ=0.235) showed smaller but nonzero correlations. Time pressure (ρ=0.153) and anxiety (ρ=0.080) showed weak day-level alignment. Person-level convergence, assessed using 15-day participant means (Table S1 in Multimedia Appendix 1), was higher than day-level estimates for most features. Social references (ρ=0.815), anger (ρ=0.751), somatic complaints (ρ=0.736), and positive emotion (ρ=0.730) showed the strongest person-level correlations. Sadness was higher at the person level (ρ=0.604) than at the day level (ρ=0.475). Anxiety showed near-zero correspondence at both levels (day-level ρ=0.080, 95% CI 0.045 to 0.115; person-level ρ=−0.022, 95% CI −0.145 to 0.101).

**Table 3.** Convergent validity between LLM-derived linguistic features and LIWC categories^a^.

Feature	Spearman ρ (95% CI)	*q* (FDR)	Zero-rate (LIWC)	Zero-rate (LLM)	Word-count sensitivity
Positive emotion	0.632 (0.608‐0.655)	<.001	0.312	0.156	Yes
Negative emotion	0.378 (0.348‐0.406)	<.001	0.052	0.012	Yes
Anxiety	0.080 (0.045‐0.115)	<.001	0.190	0.079	Yes
Anger	0.652 (0.630‐0.671)	<.001	0.777	0.405	Yes
Sadness	0.475 (0.449‐0.501)	<.001	0.858	0.444	Yes
Cognitive processing	0.367 (0.338‐0.397)	<.001	0.002	0.009	Yes
Tentativeness	0.330 (0.297‐0.361)	<.001	0.125	0.037	Yes
Self-focus	0.283 (0.251‐0.315)	<.001	0.001	0.000	No
Social references	0.786 (0.771‐0.802)	<.001	0.153	0.223	Yes
Work-related content	0.722 (0.703‐0.740)	<.001	0.138	0.097	No
Time pressure	0.153 (0.122‐0.189)	<.001	0.004	0.134	No
Somatic complaints	0.652 (0.630‐0.674)	<.001	0.421	0.217	Yes
Coping	0.235 (0.199‐0.267)	<.001	0.092	0.118	No

a Day-level Spearman rank-order correlations between large language model (LLM)–derived features and corresponding Linguistic Inquiry and Word Count (LIWC) categories. The 95% CIs were obtained via nonparametric bootstrap resampling. Two-sided asymptotic *P* values were computed and corrected for multiple testing across the 13 features using the Benjamini-Hochberg false discovery rate (FDR) procedure (*q* reported). Correlation magnitudes are interpretable using empirically derived effect-size benchmarks for psychological research, under which coefficients ≥0.30 are considered substantial. The zero rate represents the proportion of observations with a score of zero. Word-count sensitivity indicates whether concordance increased across higher word-count quartiles (yes/no).

For 9 of the 13 features, LIWC measures showed higher zero-rates than LLM-derived features; for the remaining 4 features, the ordering was reversed (Table 3). Word-count sensitivity analyses showed that concordance between the two approaches generally increased with longer texts, though not uniformly across features. Within-person stability across days (ICC-like indices; Table S1 in Multimedia Appendix 1) was higher for LLM-derived features than for LIWC counterparts for most constructs (eg, coping: 0.400 vs 0.262; cognitive processing: 0.390 vs 0.279), with exceptions for anxiety (0.320 vs 0.399), time pressure (0.259 vs 0.333), and negative emotion (0.338 vs 0.369).

Affect-related linguistic features were correlated with depressive symptoms and perceived stress (Figure S4 in Multimedia Appendix 1). Negative emotion correlated with PHQ-9 (ρ=0.32) and follow-up PSS-10 (ρ=0.41), sadness with follow-up PSS-10 (ρ=0.36), and anxiety with follow-up PSS-10 (ρ=0.30). Positive emotion was inversely correlated with PHQ-9 (ρ=−0.30) and follow-up PSS-10 (ρ=−0.28). Among personality traits, neuroticism correlated with negative emotion (ρ=0.20), and extraversion with positive emotion (ρ=0.20). Nonaffective features showed smaller correlations; time pressure (ρ=0.23) and work-related content (ρ=0.17) were positively correlated with perceived stress. Affect-related dimensions correlated more strongly with internalizing symptom measures than with personality traits.

### Domain 3: Ecological Concurrent Validity

Same-day associations between LLM-derived features and daily perceived stress were examined using GEE models with within-person and between-person decomposition (Table 4). Standardized coefficients (β) reflect the expected change in daily stress per 1-SD increase in feature intensity. At the within-person level, all features except coping were associated with daily stress after FDR correction (all *q*≤.008). The strongest positive associations were for negative emotion (β=0.68), work-related content (β=0.47), and time pressure (β=0.45). At the between-person level, most features that were significant within individuals were also significant across individuals. Self-focus, social references, and somatic complaints were not significantly associated with stress at the between-person level, nor was coping, which was also nonsignificant within persons. Replacing the exchangeable correlation structure with AR(1) yielded near-identical estimates: 25 of 26 within-person and between-person terms (96.2%) retained the same FDR-corrected significance, and the maximum absolute change in any standardized coefficient was 0.051 (within-person *r*=0.998, between-person *r*=0.997).

**Table 4.** Associations between LLM^a^-derived linguistic features and daily stress: within-person and between-person effects^b^.

Feature	Within-person			Between-person		
	β (95% CI)	*P* value	*q* value	β (95% CI)	*P* value	*q* value
Positive emotion	−0.43 (–0.51 to –0.36)	<.001	<.001	−0.24 (–0.40 to –0.07)	.004	.006
Negative emotion	0.68 (0.60 to 0.77)	<.001	<.001	0.57 (0.41 to 0.72)	<.001	<.001
Anxiety	0.32 (0.25 to 0.38)	<.001	<.001	0.37 (0.20 to 0.54)	<.001	<.001
Anger	0.36 (0.28 to 0.43)	<.001	<.001	0.30 (0.15 to 0.45)	<.001	<.001
Sadness	0.29 (0.22 to 0.37)	<.001	<.001	0.32 (0.16 to 0.47)	<.001	<.001
Cognitive processing	0.22 (0.15 to 0.29)	<.001	<.001	0.21 (0.06 to 0.36)	.006	.008
Tentativeness	0.12 (0.06 to 0.18)	<.001	<.001	0.21 (0.05 to 0.36)	.008	.010
Self-focus	0.11 (0.04 to 0.17)	.002	.003	0.11 (–0.03 to 0.25)	.13	.147
Social references	0.23 (0.16 to 0.30)	<.001	<.001	0.09 (–0.07 to 0.25)	.29	.315
Work-related content	0.47 (0.38 to 0.55)	<.001	<.001	0.32 (0.17 to 0.47)	<.001	<.001
Time pressure	0.45 (0.37 to 0.53)	<.001	<.001	0.50 (0.37 to 0.63)	<.001	<.001
Somatic complaints	0.09 (0.03 to 0.15)	.006	.008	0.00 (–0.14 to 0.13)	.99	.986
Coping	−0.06 (–0.11 to 0.00)	.07	.079	0.04 (–0.13 to 0.22)	.64	.663

a LLM: large language model.

b Estimates from generalized estimating equation models with an exchangeable correlation structure (n=279 participants; 3556 person-days). Each linguistic feature was decomposed into within-person (person-mean-centered daily deviations) and between-person (person-level mean) components, and the two components were entered simultaneously into the model. Models were adjusted for age, sex, marital status, religious affiliation, living arrangement, alcohol use, smoking status, nonworking days, assessment day, baseline Patient Health Questionnaire-9 (PHQ-9) score, Big Five personality traits, and daily word count. β denotes the standardized regression coefficient corresponding to a 1-SD increase in the linguistic feature. *q* values were corrected across the 13 features within each component using the Benjamini-Hochberg false discovery rate (FDR) procedure. Sensitivity analysis using a first-order autoregressive correlation structure yielded near-identical estimates: 25 of 26 terms (96.2%) retained the same FDR-corrected significance, the maximum absolute change in any standardized coefficient was 0.051, and β correlations between the two structures were *r*=0.998 (within-person) and *r*=0.997 (between-person).

### Domain 4: Incremental Explanatory Value

In the 4-arm comparison (baseline, baseline plus lexicon features, baseline plus LLM-derived features, and baseline plus both), the baseline model explained 13.2% of the variance in daily perceived stress (Table 5). Adding LIWC features alone yielded a modest increase to 16.9% (gain: 3.7 percentage points), whereas adding LLM-derived features increased explained variance to 30.4% (gain: 17.2 percentage points). The full model containing both feature sets reached 31.3%, only 0.9 percentage points above the LLM-only model. The incremental gain attributable to LLM-derived features was therefore approximately 4.6 times larger than the gain attributable to LIWC features alone, and adding LIWC on top of LLM features provided minimal further explanatory value. The extended models also showed lower MAE and RMSE than the baseline model across cross-validation folds.

**Table 5.** Incremental model performance for predicting daily perceived stress^a^.

Model	*R*^2^, mean (SD)	MAE^b^, mean (SD)	RMSE^c^, mean (SD)	Δ*R*^2^ vs baseline
Baseline	0.132 (0.045)	1.625 (0.102)	2.027 (0.119)	Reference
Baseline+LIWC features	0.169 (0.037)	1.577 (0.104)	1.984 (0.126)	+0.037
Baseline+LLM features	0.304 (0.042)	1.406 (0.109)	1.816 (0.130)	+0.172
Baseline+LIWC+LLM features	0.313 (0.039)	1.394 (0.118)	1.804 (0.129)	+0.181

a Values represent mean (SD) across 5 participant-level cross-validation folds. The baseline model included demographic variables, baseline Patient Health Questionnaire-9 (PHQ-9) score, Big Five personality traits, and daily word count. Linguistic Inquiry and Word Count (LIWC) and large language model (LLM) feature blocks each comprised the 13 corresponding linguistic features entered with within-person and between-person decomposition. The incremental gain from adding LIWC features alone (+0.037) was small; adding LLM features yielded a substantially larger gain (+0.172), and adding LIWC features on top of LLM features provided minimal additional improvement (+0.009).

b MAE: mean absolute error.

c RMSE: root mean square error.

### Domain 5: Expert Evaluation

#### Blinded Comparison of LLM-Derived and LIWC-Derived Feature Profiles

In a method-blinded evaluation of 104 transcripts sampled for maximal method discordance, 3 experts (1 psychiatrist and 2 clinical psychologists) independently compared deidentified feature profiles from LLM-derived and LIWC-based extraction. Across 312 decisions, LLM-derived profiles were preferred in 83.7% of cases (261/312), LIWC-based profiles in 13.1% (41/312), and neither was preferred in 3.2% (10/312) (Figure 1A). Individual expert preference rates ranged from 76.9% (80/104) to 87.5% (91/104). By majority vote (≥2 of 3 experts), LLM was preferred in 90.4% of cases (94/104), with unanimous agreement in 63.5% (66/104). Excluding ties, a 2-sided binomial test against equal preference gave *P*=2.4×10^–40^ (261 of 302 nontied decisions favored LLM, 86.4%). Interrater agreement was Gwet AC1=0.728 (substantial agreement). Fleiss κ was 0.170, reflecting the kappa paradox under skewed marginal distributions: when one option is preferred in most cases (here 261/312, 83.7%), expected chance agreement under κ is inflated and the achievable κ range is compressed.

LLM preference increased across word-count quartiles and stress severity bins (Figure 1B,C). LLM preference rose from 74.4% (58/78; Q1: ≤58 words) to 91% (71/78; Q4: ≥132 words) across word-count quartiles, and from 77.1% (111/144; low stress, EMA≤2) to 98.2% (56/57; high stress, EMA>5) across stress severity. In the high-stress subgroup, only 1 of 57 expert decisions favored LIWC. Experts also identified the constructs most central to their stress judgments, making 618 total selections across the 104 transcripts. Six constructs accounted for 84.5% (522/618) of selections: negative emotion (141/618, 22.8%), anxiety (85/618, 13.8%), positive emotion (84/618, 13.6%), work-related content (80/618, 12.9%), somatic complaints (69/618, 11.2%), and anger (63/618, 10.2%). Expert backgrounds shaped construct emphasis: the psychiatrist prioritized affective dimensions (anxiety, positive emotion), while clinical psychologists emphasized occupational context (work-related content, somatic complaints) (Figure 2A).

**Figure 1.**
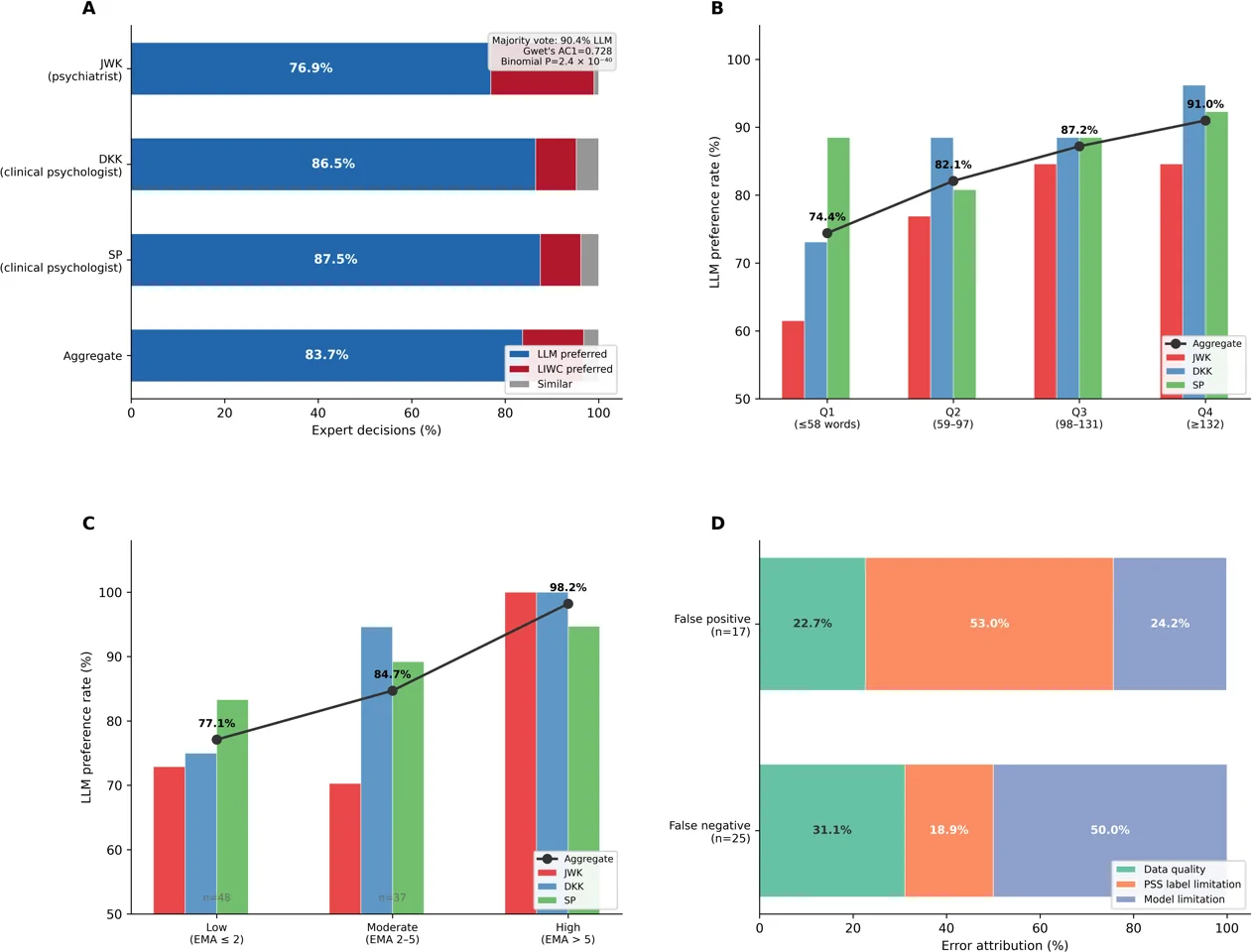
Expert evaluation of LLM-derived psycholinguistic features. Three domain experts (1 psychiatrist and 2 clinical psychologists) independently evaluated LLM-derived and LIWC-based feature profiles through blinded comparison (phase 1, n=104 transcripts) and structured error analysis (phase 2, n=42 misclassified cases). (A) Blinded A/B preference across 312 independent expert decisions. LLM-derived profiles were preferred in 83.7% (261/312) of decisions (binomial *p*=2.4×10^–40^; Gwet AC1=0.728). (B) LLM preference rate by word-count quartile, showing a monotonic increase from 74.4% (58/78; Q1: ≤58 words) to 91% (71/78; Q4: ≥132 words). Bars represent individual expert rates; the black line indicates the aggregate preference. (C) LLM preference rate by EMA-reported stress severity. Preference for LLM-derived profiles increased from 77.1% (111/144; low stress) to 98.2% (56/57; high stress), with only 1 of 57 expert decisions favoring LIWC in the high-stress subgroup. (D) Expert attribution of misclassification causes for false positive (n=17) and false negative (n=25) cases at the PSS≥19 threshold (multiple response; 156 total selections). False positives were predominantly attributed to PSS label limitations (35/66, 53%), whereas false negatives were predominantly attributed to model limitations (45/90, 50%). Overall, 60.9% (95/156) of error attributions were attributed to nonmodel factors (data quality and PSS label limitations). EMA: ecological momentary assessment; LIWC: Linguistic Inquiry and Word Count; LLM: large language model; PSS: Perceived Stress Scale.

**Figure 2.**
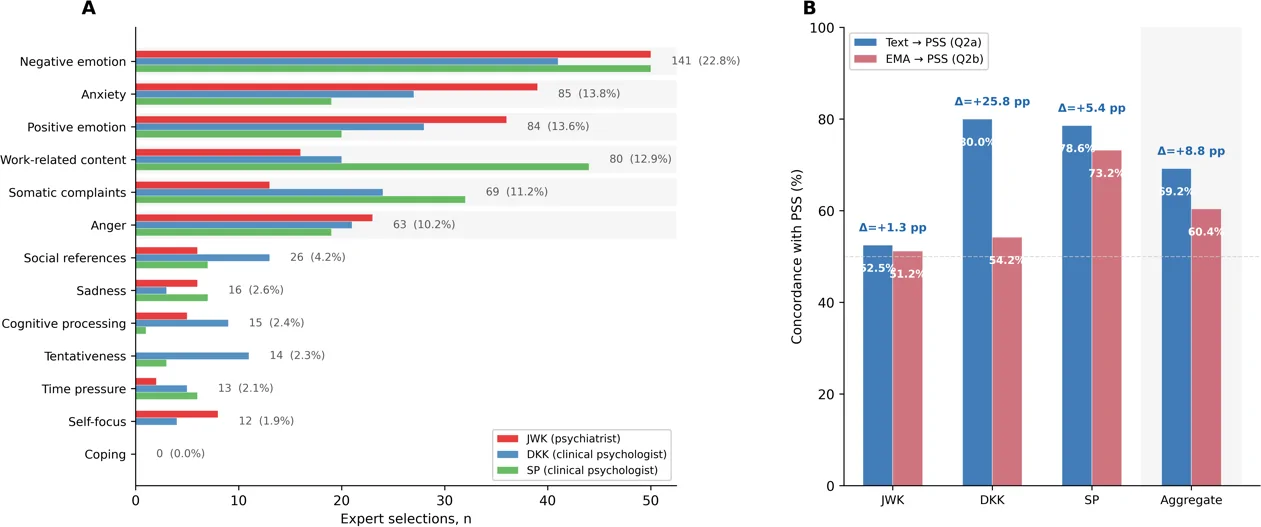
Construct centrality and text-versus-EMA concordance in expert evaluation. (A) Constructs identified by 3 domain experts as most central to clinical stress judgment (618 total selections across 104 transcripts, up to 2 constructs selected per transcript per expert). Six constructs, namely negative emotion, anxiety, positive emotion, work-related content, somatic complaints, and anger, accounted for 84.5% (522/618) of all selections. The psychiatrist (JWK) prioritized affective dimensions (anxiety and positive emotion), whereas clinical psychologists (DKK and SP) emphasized occupational context (work-related content and somatic complaints). (B) Concordance of text-based (Q2a) versus EMA-based (Q2b) trajectory assessments with retrospective PSS scores among 42 misclassified cases, using valid-response denominators (excluding indeterminate ratings). All 3 experts rated text-to-PSS concordance equal to or higher than EMA-to-PSS concordance (aggregate Δ=+8.8 percentage points), with the largest gap observed for DKK (+25.8 percentage points). EMA: ecological momentary assessment; PSS: Perceived Stress Scale.

#### Structured Error Analysis

In the second phase, the 3 experts reviewed all 42 cases misclassified by the combined baseline+LLM model at the high-stress threshold (PSS-10≥19). For each case, experts received the 15-day transcripts, daily EMA trajectory, LLM feature summary, and actual versus predicted labels. When comparing text-based and EMA-based trajectories against retrospective PSS-10, all 3 experts rated text-based trajectories as showing equal or greater concordance than EMA-based trajectories (Figure 2B). Aggregate valid-response concordance was 69.2% (74/107) for text-based versus 60.4% (64/106) for EMA-based trajectories (Δ=+8.8 percentage points). The gap was largest for one clinical psychologist (+25.8 percentage points), who frequently judged that EMA ratings underrepresented the stress level reflected in the narrative text.

Across 156 expert selections (multiple-response coding), 95 (60.9%) of attributions pointed to nonmodel factors: PSS-10 label uncertainty (52/156, 33.3%) and data quality limitations (43/156, 27.6%); model-related factors accounted for 39.1% (61/156) (Figure 1D). Error profiles differed by misclassification type. For false positives (n=17), the dominant attribution was PSS-10 label limitation (35/66, 53% of attributions). For false negatives (n=25), model limitations were predominant (45/90, 50% of attributions), with experts identifying underrecognition of defensive positivity and undifferentiated distress expression as specific gaps. Among misclassified cases, LLM summary metrics were judged informative in 72.2% (91/126) of expert evaluations (yes+partial).

### Predictive Validity for Subsequent Elevated Stress

Adding LLM features to baseline predictors improved discrimination at the higher threshold (Table 6). At PSS-10≥17, the baseline AUROC was 0.747 and the +LLM model reached 0.758; this difference of 0.011 lies within the between-fold standard deviation and is not interpreted as an improvement. The +LIWC model showed no improvement at this threshold (0.731). At PSS-10≥19, the +LLM model reached AUROC 0.854 from a baseline of 0.828 (Brier score from 0.177 to 0.146), while the +LIWC model again did not exceed the baseline (0.820). As stand-alone predictor sets, LIWC features alone yielded near-chance discrimination at the lower threshold and only modest discrimination at the higher one (AUROC 0.578 at ≥17, 0.664 at ≥19), whereas LLM features alone reached AUROC 0.691 and 0.752 at the two thresholds. Across both outcomes, the predictive contribution of LLM-derived features was not reproduced by adding LIWC features alone.

**Table 6.** Predictive performance for elevated stress at follow-up: comparison of baseline, lexicon-based, and LLM-based feature sets^a^.

Threshold and model	AUROC^b^, mean (SD)	AUPRC^c^, mean (SD)	Brier score, mean (SD)
PSS^d^≥17			
Baseline	0.747 (0.086)	0.729 (0.086)	0.226 (0.019)
LIWC only	0.578 (0.049)	0.554 (0.024)	0.246 (0.004)
LLM only	0.691 (0.084)	0.678 (0.082)	0.235 (0.014)
Baseline+LIWC	0.731 (0.108)	0.715 (0.094)	0.216 (0.029)
Baseline+LLM	0.758 (0.100)	0.755 (0.098)	0.201 (0.033)
Baseline+LIWC+LLM	0.740 (0.102)	0.732 (0.103)	0.211 (0.035)
PSS≥19			
Baseline	0.828 (0.076)	0.658 (0.098)	0.177 (0.042)
LIWC only	0.664 (0.025)	0.438 (0.028)	0.248 (0.001)
LLM only	0.752 (0.039)	0.572 (0.080)	0.204 (0.019)
Baseline+LIWC	0.820 (0.068)	0.688 (0.107)	0.165 (0.037)
Baseline+LLM	0.854 (0.033)	0.729 (0.034)	0.146 (0.026)
Baseline+LIWC+LLM	0.859 (0.052)	0.733 (0.073)	0.140 (0.031)

a Values represent mean (SD) across 5 participant-level cross-validation folds. The baseline model included demographic variables, baseline Patient Health Questionnaire-9 (PHQ-9) score, Big Five personality traits, and daily word count. Linguistic Inquiry and Word Count (LIWC) only and large language model (LLM) only models included LIWC or LLM features, respectively, together with daily word count, but no demographic or psychological covariates. Combined models add the corresponding feature blocks to the baseline.

b AUROC: area under the receiver operating characteristic curve.

c AUPRC: area under the precision-recall curve.

d PSS: Perceived Stress Scale.

### Moderation Analyses

Of 156 tested interactions, 4 survived correction, and depressive symptom severity accounted for 3 of them. Among participants with clinically significant depressive symptoms (PHQ-9≥10), the between-person association between positive emotion and stress was more strongly inverse (β=−0.57, 95% CI −0.90 to −0.23; *q*=.013), the between-person association for negative emotion was stronger (β=0.45, 95% CI 0.14-0.76; *q*=.038), and the within-person association for work-related content was stronger (β=0.37, 95% CI 0.15-0.59; *q*=.013). Among the personality traits, extraversion strengthened the between-person association for work-related content (β=0.25, 95% CI 0.11-0.39; *q*=.015). All tested interactions are given in Table S3 in Multimedia Appendix 1.

## Discussion

### Principal Findings

The 5 domains were applied to 15 days of daily narratives from 279 call center workers. Extraction was repeatable [ICC(2,*k*)=0.987]; convergence with the lexicon was construct-dependent (ρ=0.08 to 0.79); the features tracked same-day stress within individuals (β=0.68); they raised explained variance from 13.2% to 30.4%, against 16.9% for the lexicon alone; and among transcripts sampled for maximal method discordance, experts blinded to method preferred the LLM-derived profile in 83.7% (261/312) of decisions. Three properties emerged that a prediction-oriented evaluation would not have examined. Within-person and between-person associations diverged, so a feature that ranks people need not track change inside one. When experts adjudicated the misclassified cases, 53% (35/66) of false-positive attributions pointed to limits of the PSS-10 label rather than the model, and text-based trajectories were judged at least as concordant with the retrospective scale as the daily numeric ratings. And the same feature carried different meaning across people: the inverse association between positive emotion and stress was stronger under clinically significant depressive symptoms, and extraversion moderated the between-person association for work-related content. None of the three is visible in a discrimination statistic.

### Comparison With Prior Work and Interpretation

Reliability was high across all 13 features, with small absolute measurement error even at low base rates. Convergence with LIWC varied across constructs: strong for those with explicit lexical markers and weak for anxiety and time pressure. Two factors plausibly contribute: LIWC was applied to machine-translated English, which can degrade convergence for culturally specific expression, and anxiety in Korean is often hedged or situationally embedded rather than lexically marked. Anxiety nonetheless showed a within-person association with daily stress and moderate intercorrelations with cognitive processing and tentativeness, supporting its construct interpretation through nonlexicon evidence.

LLM features showed lower zero rates than LIWC for most, but not all, constructs. Correlation magnitudes in both methods fall in the small range under the benchmarks stated in the Methods section, and the claims made here do not rest on their size. Close agreement across all 13 constructs would make the model a reimplementation of the lexicon; a range this wide indicates that the two methods measure different things. The two approaches are complementary: LIWC provides transparent quantification for constructs with explicit lexical markers, while LLM-based extraction covers those whose expression depends on contextual interpretation. A fixed dictionary cannot be revised without rebuilding it, whereas the prompt can: the construct set and the rater role are specified in text and can be changed without retraining. The configuration used here is workable rather than optimized. Prior psychometrically oriented LLM work has evaluated LLM benchmarks [16], characterized LLMs as test subjects [18,44], or examined individual measurement properties such as convergent validity [2-4]. The present study integrates these threads by applying a multidomain evaluation, spanning repeatability, convergent and ecological validity, incremental comparison, and expert interpretability, to LLM-derived measurements of human psychological states tracked longitudinally.

### What Measurement-Oriented Evaluation Reveals

The present framework builds on recent evidence that LLMs can assess stable personality traits from open-ended text with psychometric validity comparable to established benchmarks [1]. That work demonstrated that LLMs can serve as psychometric instruments; the present study addresses what becomes visible when LLM-derived outputs are subjected to the full set of measurement criteria rather than evaluated primarily through convergence with self-report. The measurement-oriented framework applied here surfaces phenomena not typically reported under prediction-oriented evaluation: within-person state variation that diverges from between-person patterns, incremental explanatory value localized to LLM-derived features in a direct 4-arm comparison with a lexicon-based alternative, and structured error attribution revealing that a substantial share of apparent misclassifications reflect reference-label limitations. The application to state-level constructs, which fluctuate within individuals and require the instrument to distinguish true change from noise across repeated occasions, represents a more demanding test of these measurement properties than trait-level assessment.

The PSS-10 lacks consensus clinical thresholds; the cutoffs used here (≥17 and ≥19) are pragmatic analytic choices. Optimizing binary classification against an imperfect label risks fitting noise in the reference rather than capturing the construct, and end-to-end classification additionally sacrifices dimension-level interpretability and couples the model tightly to one population and labeling scheme. Measurement-oriented extraction is therefore better suited when the reference standard itself is uncertain.

Within-person and between-person effects diverged for several features. Positive emotion showed a stronger within-person than between-person association with stress, indicating that its stress relevance lies more in situational fluctuation than in stable disposition. Coping did not show consistent associations, possibly because coping efforts both arise from and reduce stress. Adding LLM features to baseline predictors improved discrimination and reduced Brier scores at the higher stress threshold. The 4-arm continuous-outcome comparison localized this gain to LLM-derived features rather than to linguistic features in general: the lexicon alone added little, the LLM features added substantially more, and the lexicon on top of the LLM features yielded only marginal further improvement. LLM-based extraction therefore appears to subsume most of the construct-relevant signal that lexicon matching captures, while drawing on contextual cues that fixed dictionaries cannot encode.

Four feature-stress associations varied by depressive symptom severity or personality, 3 of them by depressive symptom severity. The inverse between-person association between positive emotion and stress was stronger among individuals with clinically significant depressive symptoms (PHQ-9≥10), the between-person association for negative emotion was correspondingly stronger in the same subgroup, and the within-person association between work-related content and stress was likewise stronger. Extraversion strengthened the between-person association between work-related content and stress. The same linguistic feature can therefore carry different stress relevance across individuals, complicating uniform interpretation of language-based signals in heterogeneous populations.

Three lines of expert evidence converge. First, LLM-derived profiles were preferred over LIWC profiles in most blinded comparisons, with preference rising at higher word counts and stress severity; the advantage of contextual extraction grew with richer and more affectively loaded input. Second, text-based stress trajectories were judged equally or more concordant with retrospective PSS-10 than EMA trajectories, with experts noting that EMA ratings appeared to underrepresent the stress evident in the narrative. Prompted linguistic responses therefore captured aspects of perceived stress that brief numeric ratings miss. Third, PSS-10 label limitation was the dominant attribution for false positives, while model limitation predominated for false negatives. A meaningful share of apparent misclassifications thus reflected limitations of the dichotomized label rather than failures of the linguistic measure. This pattern is not unique to the PSS-10; any evaluation that treats an imperfect self-report scale as ground truth risks misattributing criterion limitations to the tool under evaluation, inflating apparent error rates and obscuring valid measurement signal. Experts also identified construct boundaries (defensive positivity, undifferentiated distress, and multifunctional tentativeness) that could be addressed through prompt revision rather than model retraining; whether such modifications preserve measurement properties would require separate evaluation.

### Multimodel Robustness

Multimodel evaluation tested whether these properties are model-specific. Re-extracting features with a second, independently developed model under an otherwise identical configuration yielded high cross-model agreement and near-identical convergent validity with LIWC. All but one of the 13 features retained a significant within-person association with daily stress. A systematic mean offset reflected model-level calibration rather than construct disagreement; rank ordering was preserved.

Reduced agreement for self-focus alone plausibly reflects differences in handling implicit subjects in Korean pro-drop syntax. Measurement properties were therefore not specific to a single model (Tables S4-S6 in Multimedia Appendix 1). The convergence of high extraction repeatability and agreement between two independently developed model families is consistent with stable internal representations driving these outputs. A recent interpretability study showed that an LLM develops internal representations of emotion concepts that causally influence model outputs under targeted intervention [45]. Whether such representations underlie the measurement properties observed here cannot be determined from behavioral data alone; direct tests through activation steering or emotion-vector intervention could address this question.

A parallel analysis retranslated all transcripts with an independently developed model and reran the lexicon pipeline. The published pipeline reproduced exactly, and of 54 compared quantities, 13 moved beyond the preset tolerance, all on the lexicon side; the baseline+LLM arm was unchanged (Δ*R*^2^=0.0005), and the combined arm moved only through its lexicon block (Table S7 in Multimedia Appendix 1). We did not compare the two substitutions head to head, but in both it was the lexicon pathway that moved.

### Considerations for Other Constructs and Populations

These findings support continued evaluation of LLM-based linguistic measurement as a complement to established screening tools, intended for longitudinal signal monitoring rather than stand-alone diagnosis. The input here was elicited through stress-relevant prompts rather than passively captured speech, a limitation discussed further below. One practical advantage relevant to non-English contexts is that LLM-based extraction operated directly on Korean transcripts without requiring a language-specific dictionary, whereas LIWC and similar lexicon-based tools require intermediate translation. Cross-linguistic generalizability remains to be tested empirically.

Deploying a measure of this kind in a workplace raises requirements that do not arise in research. Voice carries speaker characteristics that text does not, so retention and access rules for the original audio should be separated from those for the transcript. Only the analytic unit needs to be retained; here, the day-level feature summaries. Participation that is voluntary in form may not be voluntary in effect when the employer is the sponsor: employers should not have access to individual-level output, and participants should be able to view and withdraw their own data. The measure supports monitoring, not diagnosis, and that boundary belongs in the documents held by the IRB and by the workplace governance body.

The 5 evaluation domains applied here are not specific to occupational stress, call center workers, or the particular LLM used for extraction. Internal validity is supported by the design: repeated measures within persons, a prespecified 4-arm comparison, and cross-validated estimates. External validity is separate, and the pipeline does not transport as a whole (Textbox 1). A team moving this measure to a new construct, language, or population can follow the same order. Rerun extraction repeatability first, since it settles whether anything downstream is interpretable. Establish convergence against whatever comparator exists there, expecting the pattern rather than the values to carry over. Test the measure at the level at which it will be used, which for a state measure means within persons. Compare against the existing tool in a design that isolates the added features. Add expert review last, where resources allow.

Textbox 1. **The 5-domain framework: what transports to a new setting, what must be rederived, and what this study does not establish.**

**Transports as is**

- The 5 domains and their order
- The 4-arm design logic
- Within- and between-person decomposition
- The repeated-extraction reliability procedure
- The error-attribution coding frame

**Must be rederived** (items depend on the language, population, or comparator of the new setting)

- The 13 constructs and prompt anchors
- The mapping to lexicon categories
- The 10-item Perceived Stress Scale (PSS-10) cut point
- The moderation results
- The expert preference rate

**Not established here**

- Replication in another language or occupation
- Discriminant validity
- Consequences of acting on the measure

### Relation to Classical Validity Theory

The 5 domains were not derived from validity theory, but they map onto it (Table 7). Three facets are not covered. Discriminant validity was not a separate domain, so we cannot say these features separate perceived stress from adjacent constructs. Content validity was not established in advance by expert review; the 13 constructs were chosen to permit direct comparison with the lexicon, and because the set is defined by the prompt rather than by a fixed dictionary, it must be re-established at each extension. Consequential validity rests on one comparison of clinician preference, which speaks to acceptability. Domains 1 to 4 form a core evaluation that runs without expert involvement. Domain 5 requires experts and may be scaled down, but we recommend including it when the intended use involves clinical decision-making. The domains are ordered by increasing resource cost, not by importance. Researchers with limited resources may apply domains 1‐4 as a core evaluation and add domain 5 when feasible.

**Table 7.** Correspondence between the 5 evaluation domains and facets of validity.

Domain^a^	Validity facet	Evidence in this study
Extraction repeatability	Precondition for reliability	ICC(2,*k*)=0.987^b^
Convergent validity	External (convergent)	ρ=0.08 to 0.79
Ecological concurrent validity	Substantive and external	Within-person β=0.68
Incremental value	External (incremental)	*R*^2^=13.2% to 30.4%
Expert evaluation	Content and consequential	261/312 (83.7%) preference; error attribution

a Domains are listed in the order applied, which is also the order of increasing resource cost.

b ICC: intraclass correlation coefficient.

### Limitations

Several limitations warrant consideration. First, the linguistic input consisted of voice narratives elicited through time-specific open-ended prompts, not passively captured speech. Whether comparable properties hold for passive audio capture, brief conversational fragments, or other spontaneous daily speech is untested. In addition, linguistic features were extracted from transcripts concatenated at the day level rather than from separate morning, midday, and evening segments, and stress ratings were similarly aggregated into day-level summaries. These analytic choices preclude examination of finer-grained temporal correspondence between linguistic expression and within-day stress fluctuations. Second, LIWC-based convergent validity used machine-translated English text because no validated Korean-language LIWC dictionary is currently available; an earlier Korean adaptation exists but is no longer maintained or distributed. The two approaches therefore operated on different language inputs: LIWC on GPT-translated English and LLM features on the original Korean. Translation likely introduced information loss into the LIWC pathway, which may have attenuated some convergence estimates. Development of native-language lexicon-based benchmarks, following established cross-cultural psychometric methods [46], would remove this asymmetry but was beyond the scope of the present study. A sensitivity analysis using a second, independently developed translation model is reported under Multimodel Robustness.

Third, the study was conducted within a single occupational group, which reduces heterogeneity but limits transportability to other occupations and cultural settings; replication across diverse industries and languages is needed before broad implementation. Fourth, although two LLMs (GPT-5.2 and Gemini Flash) were evaluated under one prompt configuration, broader generalization across additional models, prompt variants, and training corpora remains untested. Fifth, the 15-day observation window may not capture longer-term trajectories. Loss to follow-up at the second study visit accounted for 165 of 207 excluded participants. Although baseline characteristics are available for these individuals, the absence of follow-up PSS-10 data precludes assessment of whether attrition was associated with the primary outcome, and the analytic sample should be interpreted as those completing the full 15-day protocol with adequate linguistic content. Sixth, predictive validity was assessed against follow-up PSS-10 thresholds. The PSS-10 is retrospective, low-frequency, and conceptually distant from real-time stress processes, which limits practical interpretation of the predictive gains. More proximal or behavioral outcomes (eg, absenteeism, health care use, sleep disturbance, clinician-rated burnout) would more directly support claims of clinical utility.

### Conclusions

Language-based mental health measures are usually evaluated as predictors, with a self-report score as the target. Prediction is not where these models are strongest, and that target records as error the properties reported here. Treating the output as a measure changes what must be shown before use: that extraction is reproducible, that convergence and divergence follow an interpretable pattern, that the measure holds at the level where it will operate, that it adds beyond an existing tool, and that experts can say what it got wrong and why. The present cohort is one test bed for that program; what carries over is the set of domains, not the 13 constructs or the population. For clinical use, this places the measure alongside existing screening instruments for longitudinal monitoring rather than in place of them, and it means that error rates computed against a self-report criterion may overstate the failures of the measure under evaluation. Whether a given LLM-derived measure is fit for use is a psychometric question, and it should be answered with psychometric evidence.

## Supplementary material

10.2196/102768 Multimedia Appendix 1 Study procedures, large language model (LLM) prompt specification, and expert evaluation materials.

## Abbreviations

AUROC: area under the receiver operating characteristic curve
BFI-10: Big Five Inventory-10
CV: coefficient of variation
EMA: ecological momentary assessment
FDR: false discovery rate
GEE: generalized estimating equations
ICC: intraclass correlation coefficient
IRB: institutional review board
LIWC: Linguistic Inquiry and Word Count
LLM: large language model
MAE: mean absolute error
PHQ-9: Patient Health Questionnaire-9
PSS-10: 10-Item Perceived Stress Scale
RMSE: root mean square error
