# Supplementary material for: Evaluating Large Language Model–Derived Outputs as Psychological Measures: A Five-Domain Psychometric Framework With Application to Daily Stress
Source: J Med Internet Res. 2026 Oct 15;28:e102768. doi: 10.2196/102768 (PMC13671668; doi:10.2196/102768)
Supplement: Supplementary file 1 [file jmir-v28-e102768-s001.docx]

**Multimedia Appendix 1**

**Study Procedures, LLM Prompt Specification, and Expert Evaluation Materials**

This supplement provides the study procedures, the complete prompt configuration used for LLM-based psycholinguistic feature extraction, and the expert evaluation materials, as referenced in the main Methods section. All prompts were composed in English; the input transcripts were Korean. Prompts and configuration were held constant across all 3,556 person-day extractions and across the multi-model robustness check using Gemini 2.5 Flash (Tables S4-S6).

**S1. Application specification**

The ecological momentary assessment application was developed for this study and ran on Android and iOS. Over two weeks, participants completed an EMA survey and a voice recording three times a day, at 08:00, 13:00, and 18:00, giving 15 study days when the first day is counted. The response window for each session was 06:30 to 09:30, 11:30 to 14:30, and 17:30 to 20:30; responses outside the window were blocked. A push notification was sent at the start of each session. Voice responses were recorded at a sampling rate of 8 kHz. Survey responses and audio files were transmitted over an encrypted connection to a study server, where access was restricted to the research team.

**S2. Transcription rules**

All voice responses were transcribed manually in Vrew by trained annotators. Personally identifiable information was removed during transcription. Disfluencies and non-content speech were excluded, including filler words (for example, “um”, “uh”), technical remarks (for example, “recording now”), speech from other individuals, and non-speech vocalizations unrelated to the participant. Within-speech pauses, laughter, nasal vocalization, and participant-produced background noise were retained. Transcripts were standardized using conventional Korean orthography with appropriate spacing and sentence-final punctuation, and numbers and foreign words were converted into Korean text.

**S3. Prompt design principles**

The prompt design followed three established principles from prior work on structured prompting for domain-specific extraction. First, recent evidence shows that explicit role assignment combined with structured task definitions and category guidelines outperforms unstructured prompting for clinical text extraction tasks. Second, integration of domain expertise with explicit category definitions and few-shot lexical examples has been shown to improve consistency and reduce hallucinations in clinical entity extraction, particularly under native-language input conditions. Third, structured prompting frameworks that specify output schema, scoring anchors, and uncertainty handling have been shown to substantially improve reproducibility of LLM-based annotations across diverse evaluation benchmarks.

Building on these principles, the system and user prompts were designed to satisfy four task-specific constraints: (1) the model functions as a psycholinguistic rater grounded in established LIWC categories, (2) scoring is conservative and reproducible, (3) Korean linguistic features (notably pro-drop syntax) are explicitly accommodated, and (4) outputs are structured JSON with a fixed key set to enable downstream analysis.

Prompts were iteratively refined through preliminary testing on a convenience subset of transcripts not included in the analytic sample. Refinement focused on three issues identified during pilot extraction: (a) drift toward inferring participant stress level from non-emotional content, (b) inflated scores under uncertainty, and (c) inconsistent treatment of Korean pro-drop sentences for self-focus scoring. The final prompt explicitly addresses each through the core principles, the scale anchors, and the Korean-specific instruction for self-focus, respectively.

The 13 features used here represent one feasible operationalization of psycholinguistic constructs commonly examined in occupational stress and mental health contexts; they are not intended as a fixed or exhaustive set. Features may be added, removed, or refined through prompt modification alone, without retraining the underlying model. The same design approach can also be applied to alternative model families (Method S7) or to different prompting paradigms (e.g., few-shot with annotated demonstrations, chain-of-thought scoring rationales). Because feature scoring is sensitive to prompt content, scale anchors, and example selection, identifying optimal feature combinations and prompt formulations for specific psychological domains is itself an empirical question that requires direct comparative evaluation. The present prompt represents a starting configuration that achieved reasonable measurement properties in this application; alternative configurations may yield improved performance for related tasks and remain to be evaluated.

**S4. System prompt**

The system prompt defines the rater role, scoring framework, scale anchors, output keys, and output constraints.

You are a PhD-level psychologist specializing in psycholinguistic analysis.

Your role is to extract quantitative, LIWC-informed linguistic features

from Korean daily diary texts written by working adults.

\## PURPOSE

This task is part of an academic research study examining relationships

between language use and self-reported stress.

You are NOT asked to evaluate stress, mental health, or well-being.

You ONLY quantify language features.

\## CORE PRINCIPLES

1. Base all scores STRICTLY on explicit linguistic content.

2. Do NOT infer psychological states beyond what is directly expressed.

3. Do NOT normalize or rescale scores based on perceived stress.

4. Be conservative, consistent, and reproducible.

5. When uncertain, prefer lower rather than higher scores.

\## SCORING FRAMEWORK

- Scores represent the relative proportion of language devoted to each category.

- All values must be continuous between 0.0 and 1.0.

- Categories are NOT mutually exclusive.

- negemo (general) and anxiety/anger/sadness (specific) are scored independently.

\## SCALE ANCHORS

- 0.0: Absent

- 0.1-0.2: Minimal

- 0.3-0.4: Moderate

- 0.5-0.6: Notable

- 0.7-0.8: Strong

- 0.9-1.0: Dominant

\## REQUIRED OUTPUT KEYS

posemo, negemo, anxiety, anger, sadness,

cogproc, tentativeness,

self_focus, social,

work, time_pressure,

somatic, coping

\## OUTPUT CONSTRAINTS

- Output MUST be a single valid JSON object.

- No explanations or text outside JSON.

- All values must be numeric floats.

**S5. User prompt template**

The user prompt provides category definitions with lexical examples and the day-level concatenated transcript. Examples in the actual prompt were provided in Korean to anchor scoring decisions in language-appropriate cues; in the version reproduced below, Korean lexical examples are translated into English glosses for reader accessibility, with the Korean originals retained in parentheses.

Analyze linguistic features in the following Korean daily diary text.

The text may include multiple time points within the same day.

Focus only on what is explicitly written.

\## IMPORTANT: KOREAN LINGUISTIC FEATURES

Korean is a pro-drop language where subjects (especially “I”) are frequently omitted.

For self_focus, count BOTH explicit pronouns AND implied first-person references.

\## CATEGORY DEFINITIONS (LIWC-informed, Korean-adapted)

\### AFFECTIVE LANGUAGE

- posemo:

Positive emotion expressions.

Examples: good (좋다), grateful (감사), proud (뿌듯), glad (기쁘다),

relieved (다행), happy (행복), satisfied (만족), okay (괜찮다)

- negemo:

General negative emotion expressions (broad category).

Examples: hard (힘들다), stressed (스트레스), bad (나쁘다),

dislike (싫다), not good (안 좋다)

- anxiety:

Worry, fear, or uncertainty about future outcomes.

Examples: worry (걱정), anxious (불안), fearful (두렵다),

uneasy (초조), don’t know how it will turn out (어떻게 될지 모르겠다)

- anger:

Frustration, irritation, or anger.

Examples: annoyed (짜증), angry (화), heated (열받다),

frustrated (답답), pissed off (빡치다)

- sadness:

Low mood, discouragement, or helplessness.

Examples: depressed (우울), sad (슬프다), upset (속상),

bitter (서럽다), gloomy (울적)

\### COGNITIVE LANGUAGE

- cogproc:

Thinking, reasoning, or causal processing.

Examples: because (왜냐하면), due to (때문에), so (그래서),

given that (~다 보니까), thinking it over (생각해보면)

- tentativeness:

Uncertainty, hedging, or lack of confidence.

Examples: it seems (것 같다), maybe (아마),

I don’t know (모르겠다), well (글쎄)

(Score genuine uncertainty, not just polite softening.)

\### SELF & SOCIAL FOCUS

- self_focus:

First-person singular self-reference.

In Korean, the subject “I” is often omitted (pro-drop).

Count BOTH:

- Explicit pronouns:

I/me (나, 저), I-subject (내가, 제가),

I-topic (나는, 저는)

- Implied: Sentences where “I” is the understood subject

(e.g., “피곤했다” = “나는 피곤했다” [I was tired],

“출근했다” = “내가 출근했다” [I went to work])

- social:

Mentions of other people or interpersonal interactions.

Examples: colleague (동료), friend (친구), family (가족),

customer (고객), petitioner (민원인), people (사람들)

\### CONTEXTUAL LANGUAGE

- work:

Job- or work-related content.

Examples: task (업무), work (일), meeting (회의),

complaint (민원), call (통화), shift (근무),

going to work (출근), leaving work (퇴근)

- time_pressure:

Urgency or time constraints.

Examples: busy (바쁘다), no time (시간 없다),

long (오래), it dragged on (길었다), continuously (계속)

\### PHYSICAL & COPING LANGUAGE

- somatic:

Physical states, fatigue, or bodily conditions.

Examples: tired (피곤), worn out (지치다),

condition (컨디션), energy (기운), sleep (잠), body (몸)

- coping:

Coping, endurance, or self-encouragement.

Examples: hold on (버티다), endure (견디다),

did well (잘했다), good job (수고했다), wrapped up (마무리했다)

---

\## TEXT TO ANALYZE

{text}

\## OUTPUT (JSON ONLY)

The placeholder {text} was substituted with each day-level concatenated transcript at extraction time.

**S6. Output schema**

Each API call returned a single JSON object with the 13 required keys. All values were continuous floats in [0.0, 1.0]. Example output:

{

“posemo”: 0.10,

“negemo”: 0.30,

“anxiety”: 0.20,

“anger”: 0.00,

“sadness”: 0.05,

“cogproc”: 0.40,

“tentativeness”: 0.25,

“self_focus”: 0.70,

“social”: 0.15,

“work”: 0.85,

“time_pressure”: 0.45,

“somatic”: 0.30,

“coping”: 0.20

}

**S7. Configuration**

The same prompt was used for both GPT-5.2 (primary extraction) and Gemini 2.5 Flash (multi-model robustness check). API parameters were:

| Parameter | GPT-5.2 | Gemini 2.5 Flash |
| --- | --- | --- |
| Model endpoint | gpt-5.2-2025-12-11 | gemini-2.5-flash |
| Temperature | 0.0 | 0.0 |
| Output format | Structured JSON (response_format) | Structured JSON (response_mime_type) |
| Reasoning / thinking | Default reasoning_effort = none | Explicit thinking_budget = 0 |
| System prompt | Identical | Identical |
| User prompt template | Identical | Identical |
| Inter-request delay | 1.0 s | 1.0 s |
| Retry policy | Up to 2 retries on malformed JSON | Up to 2 retries on malformed JSON |

The Gemini configuration was chosen to match GPT-5.2’s default non-reasoning behavior, ensuring that the multi-model comparison reflected differences in trained model behavior rather than differences in inference-time reasoning depth. Primary extraction used approximately 4,370 API calls: 3,570 for the main extraction and 800 for four additional replications of 200 texts each. Together with the original extraction of those 200 texts, this gives five measurements per text for the repeatability analysis. Total processing time was approximately 4.2 hours at an estimated cost of USD 18, based on token pricing of USD 1.75 per million input tokens and USD 14.00 per million output tokens. All analyses were conducted in Python 3.11 using statsmodels 0.14 for generalized estimating equations, scikit-learn 1.3 for regularized regression, and custom scripts for feature aggregation and decomposition. Extraction code is available in the accompanying repository (<https://github.com/pss-llm-psychometrics>).

**S8. Expert evaluation: sampling and power**

Transcripts were drawn from 52 strata defined by the 13 features and four word-count quartiles. Within each stratum we identified the transcripts showing the greatest discordance between the LLM-derived and lexicon-derived scores, and two were selected at random, giving 104 transcripts. The number of transcripts was set by the feasibility of in-depth expert review rather than by a formal a priori calculation. The resulting 312 binary preference decisions provided post-hoc power of 94.3% to detect a preference rate of 60% under a two-sided binomial test (alpha = 0.05). Sample size for the second-phase error analysis was set by the number of misclassified cases at the high-stress threshold (n = 42), which was not under design control. Three-rater designs are widely used in blinded expert evaluation of clinical text and remain feasible for structured review of every case.

**S9. Expert evaluation: questionnaire items and error-attribution categories**

***Phase 1, blinded A/B comparison.*** For each transcript, experts answered:

1. Which model better reflects the psychological stress expressed in the text: Model A, Model B, or are they similar?
2. What is the qualitative reasoning for your selection?
3. Which two constructs were most central to your clinical judgment?
4. What specific aspects of stress did the models miss or overestimate?

***Phase 2, structured error analysis.*** For each misclassified case, experts answered:

1. Does the fluctuation in stress perceived from reading the daily texts generally align with the daily self-reported EMA stress scores?
2. (a) Does the 15-day trajectory of the texts align with the directional change or final level of the retrospective PSS-10? (b) Does the 15-day trajectory of the EMA scores align with the directional change or final level of the retrospective PSS-10?
3. Do the LLM-derived summary metrics effectively explain the PSS-10 levels or changes?
4. What is the primary cause of the model’s predictive failure?

For question 4, the primary cause was assigned to one of three predefined categories: data limitations (for example, ambiguous texts or lack of text-EMA concordance), label limitations (for example, PSS-10 cutoff rigidity or temporal mismatch between the 15-day texts and the retrospective PSS-10), or model limitations (for example, failure of the LLM summary metrics to capture key narrative elements). Multiple responses were permitted, and a multiple-response analysis quantified the relative contribution of each factor. Prior to independent evaluation the three experts took part in a calibration session; evaluations were then conducted blinded and independently, and aggregation was performed by a researcher who took no part in the evaluation.

**Table S1.** Convergent validity and distributional comparison between LLM-derived features and LIWC-based lexical proxies.

| Feature | person-level ρ (95% CI) | q (FDR) | WC effect on ρ | ICC-like (LLM) | ICC-like (LIWC) |
| --- | --- | --- | --- | --- | --- |
| Positive emotion | 0.730 [0.662-0.785] | < .001 | * | 0.305 | 0.249 |
| Negative emotion | 0.371 [0.255-0.468] | < .001 | * | 0.338 | 0.369 |
| Anxiety | −0.022 [−0.145-0.101] | .711 | * | 0.320 | 0.399 |
| Anger | 0.751 [0.685-0.808] | < .001 | * | 0.280 | 0.208 |
| Sadness | 0.604 [0.514-0.681] | < .001 | * | 0.255 | 0.151 |
| Cognitive processing | 0.436 [0.334-0.534] | < .001 | * | 0.390 | 0.279 |
| Tentativeness | 0.446 [0.343-0.545] | < .001 | * | 0.378 | 0.240 |
| Self-focus | 0.272 [0.147-0.382] | < .001 | ns | 0.323 | 0.300 |
| Social references | 0.815 [0.756-0.859] | < .001 | * | 0.294 | 0.296 |
| Work-related content | 0.671 [0.595-0.740] | < .001 | ns | 0.277 | 0.243 |
| Time pressure | 0.310 [0.191-0.421] | < .001 | ns | 0.259 | 0.333 |
| Somatic complaints | 0.736 [0.671-0.791] | < .001 | * | 0.306 | 0.215 |
| Coping | 0.275 [0.154-0.389] | < .001 | ns | 0.400 | 0.262 |

*Note.* Person-level Spearman rank-order correlations between LLM-derived features and LIWC categories, computed using 15-day participant means (n = 279). 95% confidence intervals were obtained via nonparametric bootstrap resampling. Two-sided asymptotic p-values were computed and corrected across the 13 features using the Benjamini-Hochberg false discovery rate procedure (q reported). Word-count sensitivity (* yes; ns no) indicates whether concordance increased across higher word-count quartiles. ICC-like values represent descriptive indices of within-person stability across days and should not be interpreted as formal intraclass correlation coefficients. Anxiety showed near-zero person-level convergence (q = .71), consistent with the day-level pattern (Table 3) and discussed in the main text. LLM = large language model; LIWC = Linguistic Inquiry and Word Count.

**Table S2.** Descriptive statistics of LLM-derived linguistic features.

| Linguistic feature | First EMA assessment day | Participant-level mean across EMA period |
| --- | --- | --- |
| Positive emotion | 0.130 (0.183) | 0.244 (0.120) |
| Negative emotion | 0.479 (0.219) | 0.409 (0.126) |
| Anxiety | 0.168 (0.165) | 0.155 (0.077) |
| Anger | 0.147 (0.205) | 0.125 (0.098) |
| Sadness | 0.044 (0.101) | 0.061 (0.050) |
| Cognitive processing | 0.310 (0.163) | 0.290 (0.085) |
| Tentativeness | 0.193 (0.130) | 0.206 (0.071) |
| Self-focus | 0.539 (0.177) | 0.642 (0.076) |
| Social references | 0.223 (0.211) | 0.185 (0.091) |
| Work-related content | 0.554 (0.288) | 0.435 (0.149) |
| Time pressure | 0.170 (0.177) | 0.163 (0.077) |
| Somatic complaints | 0.126 (0.199) | 0.208 (0.119) |
| Coping | 0.105 (0.115) | 0.154 (0.073) |

*Note.* *Values are presented as mean (SD). Participant-level means were calculated by averaging daily feature scores across the EMA period.*

**Table S3.** Moderation of feature-stress associations by depressive symptom severity and personality traits: all tested interactions.

| Moderator / Feature | | Level | Beta interaction (95% CI) | SE | q-value |
| --- | --- | --- | --- | --- | --- |
| PHQ-9 ≥ 10 |  |  |  |  |  |
| Anger | | Between | 0.11 (-0.21, 0.43) | 0.16 | .646 |
| Anger | | Within | -0.03 (-0.20, 0.15) | 0.09 | .894 |
| Anxiety | | Between | 0.13 (-0.20, 0.45) | 0.17 | .633 |
| Anxiety | | Within | 0.15 (0.00, 0.30) | 0.08 | .131 |
| Cognitive processing | | Between | -0.18 (-0.56, 0.19) | 0.19 | .585 |
| Cognitive processing | | Within | 0.20 (0.04, 0.37) | 0.08 | .082 |
| Coping | | Between | -0.42 (-0.83, -0.01) | 0.21 | .131 |
| Coping | | Within | -0.02 (-0.16, 0.11) | 0.07 | .891 |
| Negative emotion | | Between | **0.45 (0.14, 0.76)** | 0.16 | **.038** |
| Negative emotion | | Within | 0.23 (0.01, 0.45) | 0.11 | .131 |
| Positive emotion | | Between | **-0.57 (-0.90, -0.23)** | 0.17 | **.013** |
| Positive emotion | | Within | -0.15 (-0.36, 0.06) | 0.11 | .392 |
| Sadness | | Between | 0.01 (-0.29, 0.31) | 0.15 | .982 |
| Sadness | | Within | -0.09 (-0.26, 0.07) | 0.08 | .508 |
| Self-focus | | Between | 0.03 (-0.33, 0.39) | 0.18 | .971 |
| Self-focus | | Within | 0.06 (-0.09, 0.22) | 0.08 | .633 |
| Social references | | Between | 0.03 (-0.41, 0.47) | 0.23 | .971 |
| Social references | | Within | 0.17 (0.01, 0.33) | 0.08 | .131 |
| Somatic complaints | | Between | -0.19 (-0.47, 0.10) | 0.15 | .445 |
| Somatic complaints | | Within | -0.00 (-0.16, 0.16) | 0.08 | .982 |
| Tentativeness | | Between | 0.18 (-0.14, 0.50) | 0.16 | .508 |
| Tentativeness | | Within | 0.18 (0.04, 0.32) | 0.07 | .070 |
| Time pressure | | Between | 0.11 (-0.21, 0.44) | 0.17 | .646 |
| Time pressure | | Within | 0.21 (-0.01, 0.42) | 0.11 | .158 |
| Work-related content | | Between | 0.15 (-0.21, 0.51) | 0.18 | .633 |
| Work-related content | | Within | **0.37 (0.15, 0.59)** | 0.11 | **.013** |
| Agreeableness | | | | | |
| Anger | | Between | -0.09 (-0.24, 0.05) | 0.07 | .606 |
| Anger | | Within | -0.01 (-0.08, 0.07) | 0.04 | .949 |
| Anxiety | | Between | -0.09 (-0.22, 0.04) | 0.06 | .606 |
| Anxiety | | Within | -0.04 (-0.10, 0.03) | 0.03 | .650 |
| Cognitive processing | | Between | -0.11 (-0.25, 0.03) | 0.07 | .585 |
| Cognitive processing | | Within | -0.05 (-0.11, 0.01) | 0.03 | .585 |
| Coping | | Between | -0.14 (-0.31, 0.02) | 0.09 | .585 |
| Coping | | Within | -0.02 (-0.07, 0.03) | 0.03 | .781 |
| Negative emotion | | Between | -0.06 (-0.17, 0.05) | 0.05 | .651 |
| Negative emotion | | Within | -0.00 (-0.09, 0.08) | 0.04 | .949 |
| Positive emotion | | Between | 0.06 (-0.09, 0.20) | 0.07 | .781 |
| Positive emotion | | Within | -0.02 (-0.10, 0.05) | 0.04 | .781 |
| Sadness | | Between | -0.02 (-0.16, 0.12) | 0.07 | .949 |
| Sadness | | Within | 0.02 (-0.07, 0.11) | 0.05 | .781 |
| Self-focus | | Between | -0.06 (-0.15, 0.02) | 0.04 | .606 |
| Self-focus | | Within | -0.06 (-0.11, -0.00) | 0.03 | .450 |
| Social references | | Between | -0.06 (-0.24, 0.12) | 0.09 | .781 |
| Social references | | Within | 0.00 (-0.05, 0.06) | 0.03 | .949 |
| Somatic complaints | | Between | -0.04 (-0.16, 0.08) | 0.06 | .781 |
| Somatic complaints | | Within | -0.01 (-0.06, 0.05) | 0.03 | .949 |
| Tentativeness | | Between | -0.08 (-0.21, 0.04) | 0.06 | .606 |
| Tentativeness | | Within | -0.06 (-0.11, -0.01) | 0.03 | .450 |
| Time pressure | | Between | -0.03 (-0.16, 0.09) | 0.07 | .781 |
| Time pressure | | Within | -0.02 (-0.11, 0.06) | 0.04 | .781 |
| Work-related content | | Between | 0.00 (-0.14, 0.14) | 0.07 | .949 |
| Work-related content | | Within | -0.03 (-0.11, 0.05) | 0.04 | .781 |
| Conscientiousness | | | | | |
| Anger | | Between | -0.03 (-0.16, 0.11) | 0.07 | .951 |
| Anger | | Within | -0.03 (-0.10, 0.05) | 0.04 | .852 |
| Anxiety | | Between | 0.01 (-0.12, 0.13) | 0.07 | .951 |
| Anxiety | | Within | 0.01 (-0.06, 0.08) | 0.03 | .951 |
| Cognitive processing | | Between | -0.01 (-0.15, 0.13) | 0.07 | .951 |
| Cognitive processing | | Within | -0.07 (-0.13, -0.01) | 0.03 | .322 |
| Coping | | Between | -0.02 (-0.18, 0.14) | 0.08 | .951 |
| Coping | | Within | -0.03 (-0.09, 0.02) | 0.03 | .852 |
| Negative emotion | | Between | -0.05 (-0.16, 0.05) | 0.06 | .852 |
| Negative emotion | | Within | 0.02 (-0.06, 0.11) | 0.04 | .904 |
| Positive emotion | | Between | -0.05 (-0.19, 0.08) | 0.07 | .852 |
| Positive emotion | | Within | -0.00 (-0.08, 0.08) | 0.04 | .951 |
| Sadness | | Between | -0.00 (-0.10, 0.10) | 0.05 | .951 |
| Sadness | | Within | -0.01 (-0.09, 0.08) | 0.04 | .951 |
| Self-focus | | Between | -0.06 (-0.19, 0.07) | 0.07 | .852 |
| Self-focus | | Within | 0.02 (-0.04, 0.08) | 0.03 | .855 |
| Social references | | Between | -0.06 (-0.22, 0.09) | 0.08 | .852 |
| Social references | | Within | -0.08 (-0.14, -0.02) | 0.03 | .275 |
| Somatic complaints | | Between | -0.04 (-0.17, 0.09) | 0.07 | .855 |
| Somatic complaints | | Within | 0.05 (-0.01, 0.11) | 0.03 | .802 |
| Tentativeness | | Between | -0.05 (-0.17, 0.07) | 0.06 | .852 |
| Tentativeness | | Within | -0.04 (-0.10, 0.01) | 0.03 | .802 |
| Time pressure | | Between | 0.05 (-0.07, 0.17) | 0.06 | .852 |
| Time pressure | | Within | 0.04 (-0.04, 0.13) | 0.04 | .852 |
| Work-related content | | Between | -0.07 (-0.21, 0.08) | 0.08 | .852 |
| Work-related content | | Within | -0.03 (-0.12, 0.06) | 0.05 | .852 |
| Extraversion | | | | | |
| Anger | | Between | 0.01 (-0.11, 0.13) | 0.06 | .975 |
| Anger | | Within | -0.05 (-0.11, 0.02) | 0.03 | .791 |
| Anxiety | | Between | 0.00 (-0.17, 0.18) | 0.09 | .975 |
| Anxiety | | Within | -0.01 (-0.07, 0.05) | 0.03 | .952 |
| Cognitive processing | | Between | -0.03 (-0.16, 0.11) | 0.07 | .946 |
| Cognitive processing | | Within | -0.05 (-0.11, 0.01) | 0.03 | .667 |
| Coping | | Between | 0.03 (-0.15, 0.21) | 0.09 | .946 |
| Coping | | Within | 0.01 (-0.04, 0.07) | 0.03 | .946 |
| Negative emotion | | Between | 0.03 (-0.09, 0.14) | 0.06 | .946 |
| Negative emotion | | Within | -0.03 (-0.11, 0.04) | 0.04 | .946 |
| Positive emotion | | Between | -0.03 (-0.16, 0.10) | 0.07 | .946 |
| Positive emotion | | Within | 0.00 (-0.07, 0.08) | 0.04 | .975 |
| Sadness | | Between | -0.00 (-0.14, 0.13) | 0.07 | .975 |
| Sadness | | Within | 0.05 (-0.03, 0.13) | 0.04 | .820 |
| Self-focus | | Between | -0.05 (-0.17, 0.07) | 0.06 | .946 |
| Self-focus | | Within | -0.02 (-0.08, 0.04) | 0.03 | .946 |
| Social references | | Between | 0.01 (-0.15, 0.17) | 0.08 | .975 |
| Social references | | Within | -0.08 (-0.14, -0.02) | 0.03 | .072 |
| Somatic complaints | | Between | -0.04 (-0.16, 0.09) | 0.06 | .946 |
| Somatic complaints | | Within | 0.07 (0.01, 0.14) | 0.03 | .195 |
| Tentativeness | | Between | -0.08 (-0.21, 0.05) | 0.07 | .791 |
| Tentativeness | | Within | -0.03 (-0.09, 0.02) | 0.03 | .791 |
| Time pressure | | Between | 0.04 (-0.08, 0.16) | 0.06 | .946 |
| Time pressure | | Within | 0.03 (-0.05, 0.11) | 0.04 | .946 |
| Work-related content | | Between | **0.25 (0.11, 0.39)** | 0.07 | **.015** |
| Work-related content | | Within | -0.02 (-0.09, 0.05) | 0.04 | .946 |
| Neuroticism | | | | | |
| Anger | | Between | 0.08 (-0.08, 0.24) | 0.08 | .892 |
| Anger | | Within | -0.01 (-0.09, 0.07) | 0.04 | .902 |
| Anxiety | | Between | 0.04 (-0.10, 0.18) | 0.07 | .892 |
| Anxiety | | Within | 0.02 (-0.04, 0.09) | 0.04 | .892 |
| Cognitive processing | | Between | 0.05 (-0.09, 0.20) | 0.07 | .892 |
| Cognitive processing | | Within | 0.03 (-0.04, 0.10) | 0.04 | .892 |
| Coping | | Between | 0.05 (-0.10, 0.20) | 0.08 | .892 |
| Coping | | Within | 0.01 (-0.05, 0.07) | 0.03 | .902 |
| Negative emotion | | Between | 0.05 (-0.07, 0.17) | 0.06 | .892 |
| Negative emotion | | Within | -0.01 (-0.10, 0.08) | 0.05 | .913 |
| Positive emotion | | Between | -0.11 (-0.24, 0.02) | 0.07 | .892 |
| Positive emotion | | Within | -0.00 (-0.09, 0.08) | 0.04 | .972 |
| Sadness | | Between | -0.03 (-0.18, 0.11) | 0.07 | .902 |
| Sadness | | Within | -0.07 (-0.14, 0.00) | 0.04 | .892 |
| Self-focus | | Between | 0.06 (-0.06, 0.18) | 0.06 | .892 |
| Self-focus | | Within | 0.01 (-0.05, 0.08) | 0.03 | .902 |
| Social references | | Between | 0.10 (-0.05, 0.26) | 0.08 | .892 |
| Social references | | Within | 0.02 (-0.04, 0.08) | 0.03 | .892 |
| Somatic complaints | | Between | 0.01 (-0.11, 0.13) | 0.06 | .913 |
| Somatic complaints | | Within | -0.03 (-0.10, 0.04) | 0.03 | .892 |
| Tentativeness | | Between | 0.07 (-0.08, 0.22) | 0.08 | .892 |
| Tentativeness | | Within | 0.04 (-0.01, 0.10) | 0.03 | .892 |
| Time pressure | | Between | 0.03 (-0.10, 0.15) | 0.06 | .902 |
| Time pressure | | Within | 0.01 (-0.08, 0.09) | 0.04 | .913 |
| Work-related content | | Between | 0.02 (-0.11, 0.16) | 0.07 | .902 |
| Work-related content | | Within | 0.05 (-0.03, 0.13) | 0.04 | .892 |
| Openness | | | | | |
| Anger | | Between | 0.06 (-0.08, 0.19) | 0.07 | .701 |
| Anger | | Within | 0.06 (-0.00, 0.13) | 0.04 | .625 |
| Anxiety | | Between | -0.06 (-0.20, 0.09) | 0.07 | .701 |
| Anxiety | | Within | -0.01 (-0.08, 0.05) | 0.03 | .839 |
| Cognitive processing | | Between | -0.12 (-0.27, 0.04) | 0.08 | .625 |
| Cognitive processing | | Within | -0.01 (-0.07, 0.06) | 0.03 | .900 |
| Coping | | Between | -0.12 (-0.27, 0.03) | 0.08 | .625 |
| Coping | | Within | -0.01 (-0.07, 0.04) | 0.03 | .802 |
| Negative emotion | | Between | 0.04 (-0.09, 0.17) | 0.07 | .777 |
| Negative emotion | | Within | 0.04 (-0.05, 0.12) | 0.04 | .701 |
| Positive emotion | | Between | 0.03 (-0.11, 0.16) | 0.07 | .839 |
| Positive emotion | | Within | -0.06 (-0.14, 0.02) | 0.04 | .625 |
| Sadness | | Between | 0.01 (-0.10, 0.13) | 0.06 | .876 |
| Sadness | | Within | 0.01 (-0.08, 0.09) | 0.04 | .901 |
| Self-focus | | Between | -0.05 (-0.19, 0.09) | 0.07 | .736 |
| Self-focus | | Within | -0.03 (-0.10, 0.03) | 0.03 | .701 |
| Social references | | Between | 0.04 (-0.11, 0.18) | 0.07 | .802 |
| Social references | | Within | 0.03 (-0.04, 0.10) | 0.03 | .701 |
| Somatic complaints | | Between | -0.06 (-0.18, 0.06) | 0.06 | .701 |
| Somatic complaints | | Within | 0.04 (-0.01, 0.09) | 0.03 | .625 |
| Tentativeness | | Between | -0.07 (-0.20, 0.06) | 0.07 | .701 |
| Tentativeness | | Within | -0.05 (-0.10, -0.00) | 0.03 | .625 |
| Time pressure | | Between | -0.09 (-0.24, 0.05) | 0.08 | .701 |
| Time pressure | | Within | 0.05 (-0.04, 0.14) | 0.05 | .701 |
| Work-related content | | Between | -0.05 (-0.19, 0.10) | 0.07 | .770 |
| Work-related content | | Within | 0.04 (-0.05, 0.12) | 0.04 | .701 |

*Note.* Estimates are interaction terms from generalized estimating equation models with an exchangeable correlation structure, in which each linguistic feature was decomposed into within-person and between-person components. All 156 tested interactions are shown. Benjamini-Hochberg correction was applied separately within each moderator, across the 26 interaction terms for that moderator; terms surviving correction are in bold. Models were adjusted for the same covariates as the main analyses, and the variable serving as the moderator was excluded from the covariate set in that model. PHQ-9 = Patient Health Questionnaire-9.

**Table S4.** Cross-model agreement between GPT-5.2 and Gemini Flash.

| Feature | Day-level r (Pearson) | Day-level ρ (Spearman) | Person-level r | Person-level ρ | Mean diff (Gemini - GPT) |
| --- | --- | --- | --- | --- | --- |
| Positive emotion | 0.909 | 0.928 | 0.944 | 0.943 | -0.015 |
| Negative emotion | 0.884 | 0.912 | 0.951 | 0.946 | -0.101 |
| Anxiety | 0.854 | 0.861 | 0.939 | 0.939 | -0.068 |
| Anger | 0.881 | 0.865 | 0.947 | 0.946 | -0.049 |
| Sadness | 0.822 | 0.743 | 0.913 | 0.887 | -0.023 |
| Cognitive processing | 0.676 | 0.678 | 0.879 | 0.868 | -0.168 |
| Tentativeness | 0.710 | 0.720 | 0.888 | 0.894 | -0.095 |
| Self-focus | 0.383 | 0.308 | 0.366 | 0.314 | -0.021 |
| Social references | 0.890 | 0.919 | 0.953 | 0.953 | -0.067 |
| Work-related content | 0.878 | 0.894 | 0.941 | 0.928 | -0.166 |
| Time pressure | 0.796 | 0.794 | 0.893 | 0.903 | -0.079 |
| Somatic complaints | 0.886 | 0.910 | 0.957 | 0.953 | -0.061 |
| Coping | 0.807 | 0.826 | 0.932 | 0.935 | -0.057 |
| Median | 0.854 | 0.861 | 0.939 | 0.928 | -0.067 |

*Note.* Cross-model agreement was assessed at both day-level (n = 3,556 person-days) and person-level (n = 279 participants, using 15-day means). Pearson r and Spearman ρ are reported for each level. Mean diff is the systematic difference in feature scores (Gemini Flash minus GPT-5.2), averaged across observations. All p-values < .001 except where noted by NS in main text. The median Pearson r at day level was 0.854 (range 0.38 to 0.91), with self-focus showing the lowest agreement, plausibly reflecting differences in how each model handles implicit subjects in Korean pro-drop syntax. Both extractions used identical prompts at temperature 0; reasoning was disabled for both models (see Section S7). LLM = large language model.

**Table S5.** Convergent validity comparison: GPT-5.2 vs Gemini Flash (both vs LIWC, day-level).

| Feature | GPT-5.2 ρ | GPT-5.2 q | Gemini ρ | Gemini q | Δρ (Gemini - GPT) |
| --- | --- | --- | --- | --- | --- |
| Positive emotion | 0.632 | < .001 | 0.576 | < .001 | -0.056 |
| Negative emotion | 0.378 | < .001 | 0.394 | < .001 | +0.016 |
| Anxiety | 0.080 | < .001 | 0.062 | < .001 | -0.018 |
| Anger | 0.652 | < .001 | 0.638 | < .001 | -0.014 |
| Sadness | 0.475 | < .001 | 0.459 | < .001 | -0.016 |
| Cognitive processing | 0.367 | < .001 | 0.302 | < .001 | -0.065 |
| Tentativeness | 0.330 | < .001 | 0.319 | < .001 | -0.010 |
| Self-focus | 0.283 | < .001 | 0.294 | < .001 | +0.012 |
| Social references | 0.786 | < .001 | 0.747 | < .001 | -0.039 |
| Work-related content | 0.722 | < .001 | 0.702 | < .001 | -0.020 |
| Time pressure | 0.153 | < .001 | 0.180 | < .001 | +0.027 |
| Somatic complaints | 0.652 | < .001 | 0.647 | < .001 | -0.004 |
| Coping | 0.235 | < .001 | 0.183 | < .001 | -0.051 |
| Median | 0.378 | | 0.394 | |  |

*Note.* Day-level Spearman rank-order correlations between LLM-derived features (separately for GPT-5.2 and Gemini Flash) and corresponding LIWC categories (n = 3,556 person-days). The 95% confidence intervals are not shown for compactness but are available on request. q-values were FDR-BH corrected within each model across the 13 features. Δρ represents the difference in convergent validity between the two models (positive = Gemini stronger). Across the 13 features, Gemini’s day-level convergence was within ±0.07 of GPT-5.2’s for all features, with median ρ values nearly identical (0.394 vs 0.378). LLM = large language model; LIWC = Linguistic Inquiry and Word Count.

**Table S6.** Ecological validity comparison: within-person GEE coefficients across models.

| Feature | GPT-5.2 β_w | GPT-5.2 q | Gemini β_w | Gemini q | Δβ_w |
| --- | --- | --- | --- | --- | --- |
| Positive emotion | -0.43 | < .001 | -0.48 | < .001 | -0.05 |
| Negative emotion | 0.68 | < .001 | 0.64 | < .001 | -0.04 |
| Anxiety | 0.32 | < .001 | 0.35 | < .001 | +0.03 |
| Anger | 0.36 | < .001 | 0.43 | < .001 | +0.07 |
| Sadness | 0.29 | < .001 | 0.35 | < .001 | +0.05 |
| Cognitive processing | 0.22 | < .001 | 0.19 | < .001 | -0.03 |
| Tentativeness | 0.12 | < .001 | 0.12 | < .001 | < 0.01 |
| Self-focus | 0.11 | .003 | 0.01 | .878 | -0.10 |
| Social references | 0.23 | < .001 | 0.23 | < .001 | < 0.01 |
| Work-related content | 0.47 | < .001 | 0.47 | < .001 | < 0.01 |
| Time pressure | 0.45 | < .001 | 0.36 | < .001 | -0.09 |
| Somatic complaints | 0.09 | .008 | 0.12 | < .001 | +0.04 |
| Coping | -0.06 | .079 | -0.09 | .002 | -0.04 |

*Note.* Within-person standardized GEE coefficients for each LLM-derived feature predicting daily stress, computed separately for GPT-5.2 and Gemini Flash extractions on the same 3,556 person-days. Models adjusted for age, sex, marital status, religious affiliation, living arrangement, alcohol use, smoking status, nonworking days, assessment day, baseline PHQ-9 score, Big Five personality traits, and daily word count. q-values were FDR-BH corrected across the 13 features within each model. Eleven of 13 features were FDR-significant in both models (q < .05); coping reached significance under Gemini extraction but not GPT (GPT q = .079; Gemini q = .002), and self-focus reached significance under GPT but not Gemini (q = .003 vs .878). The four largest within-person coefficients (negemo, work, time pressure, anxiety) replicated across models with Δβ ≤ 0.09. LLM = large language model; PHQ-9 = Patient Health Questionnaire-9.

**Table S7.** Translation sensitivity: lexicon pathway under two independently developed translation models.

| Model | Largest change in R^2^ across compared quantities |
| --- | --- |
| Baseline | 0.0004 |
| Baseline + LLM features | 0.0005 |
| Baseline + lexicon features | 0.0172 |
| Baseline + both | 0.0490 |

*Note.* All transcripts were re-translated with a second, independently developed model and the lexicon pipeline was re-run. The published pipeline reproduced exactly: zero rates matched across Table 3 and all 13 categories. Of 54 compared quantities, 13 moved beyond the pre-set tolerance of 0.02, all of them on the lexicon side. Correlations between the two translation pathways ranged from 0.690 to 0.965 (median 0.868); the lowest values were function-word categories and the highest were content-word categories. Self-focus moved most, consistent with how each pathway handles subject omission in Korean. Results are limited to these two pathways and do not establish independence from translators in general.

**Figure S1. Flow chart.** Participant flow. Of 486 enrolled participants, 207 were excluded under the prespecified analytic criteria: 165 for missing follow-up PSS-10 data, 18 for invariant PSS-10 responses (total score of 0 at both time points), 15 for mean day-level transcript length below 10 words, and 9 for fewer than three completed EMA days. The final analytic sample comprised 279 participants contributing 3,556 person-days. The companion report begins from the 480 participants who contributed at least one EMA entry.

**Figure S2.** **Distributions of LLM-derived linguistic feature scores across EMA entries.** Histograms show the distributions of LLM-derived linguistic feature scores extracted from 3,556 valid EMA voice recordings. Features span affective, cognitive, and work-related domains. Visual inspection suggests that feature scores span a broad range of values across constructs, with sufficient variability for downstream analyses.

**Figure S3. Pairwise intercorrelations among LLM-derived linguistic features.** Heatmap of Spearman correlation coefficients among LLM-derived linguistic features aggregated at the participant level. Warmer colors indicate stronger positive associations, whereas cooler colors indicate negative associations. Overall correlation magnitudes were moderate, and the highest pairwise value was 0.71, supporting the interpretation that the dimensions capture related yet non-redundant constructs across affective, cognitive, and work-related domains.

**Figure S4. Convergent & Discriminant Validity Heatmap.** Heatmap showing Spearman correlation coefficients between participant-level mean LLM-derived linguistic features and clinical measures of depressive symptoms (PHQ-9), perceived stress (PSS), and personality traits assessed using the Big Five Inventory-10. Warmer colors indicate positive correlations, and cooler colors indicate negative correlations, with color intensity reflecting correlation magnitude. Asterisks denote statistical significance (*p < 0.05, **p < 0.01, ***p < 0.001). Overall, correlation patterns demonstrate theoretically coherent convergent associations with affective symptom measures and more selective relationships with personality traits, supporting construct validity.
